# Diffusion in multicomponent aqueous alcoholic mixtures

**DOI:** 10.1038/s41598-021-91727-w

**Published:** 2021-06-10

**Authors:** Gabriela Guevara-Carrion, Robin Fingerhut, Jadran Vrabec

**Affiliations:** Thermodynamics and Process Engineering, Technical University of Berlin, Ernst-Reuter-Platz 1, 10587 Berlin, Germany

**Keywords:** Mathematics and computing, Fluid dynamics, Statistical physics, thermodynamics and nonlinear dynamics

## Abstract

The Fick diffusion coefficient matrix of the highly associating quaternary mixture water + methanol + ethanol + 2-propanol as well as its ternary and binary subsystems is analyzed with molecular dynamics simulation techniques. Three of the ternary subsystems are studied in this sense for the first time. The predictive capability of the employed force fields, which were sampled with the Green–Kubo formalism and Kirkwood–Buff integration, is confirmed by comparison with experimental literature data on vapor-liquid equilibrium, shear viscosity and Fick diffusion coefficient, wherever possible. A thorough analysis of the finite size effects on the simulative calculation of diffusion coefficients of multicomponent systems is carried out. Moreover, the dependence of the Fick diffusion coefficient matrix on the velocity reference frame and component order is analyzed. Their influence is found to be less significant for the main matrix elements, reaching a maximum variation of 19%. The large differences found for the cross elements upon variation of the reference frame hinder a straightforward interpretation of the Fick diffusion coefficient matrix with respect to the presence of diffusive coupling effects.

## Introduction

Diffusion processes are ubiquitous and are thus an important research topic in many disciplines, such as physics, chemistry, biology, medicine and engineering. In fact, diffusion is the key to describe the propagation of molecular species in liquids^1^, porous materials^2^, depleted oil reservoirs^3^, solar cells^4^ or human histoid^5^. Most of these processes involve mixtures of more than two components and their modeling requires the determination of transport diffusion coefficients. Because of the nature of diffusion, experiments are laborious and time-consuming, especially for multicomponent systems which usually necessitate several experiments for each state point^6^. Further, the dimensionality of the measurement space follows a power law increase with each additional component, making a comprehensive measurement of multicomponent systems exhaustive and expensive^7^. It is thus not surprising that experimental data on multicomponent diffusion are scarce^8,9^ and manifest in many cases the bottleneck for understanding, modeling and designing realistic processes^6^. Although the investigation of diffusion started in the 1850s with the work of Graham^10^ and Fick^11^, experimental data on transport diffusion coefficients for only around 200 ternary mixtures^6,9^ and less than 20 quaternary mixtures^8,12^ have been reported in the literature. Obviously, experimental measurements alone are not able to satisfy the growing need for accurate transport diffusion coefficients, particularly for liquids constituted by many components.

The underlying physical phenomena to mass transport in multicomponent mixtures are quite complex and still not well understood because of the presence of coupling effects, which may lead to uphill diffusion^13^. For instance, the description of the isothermal–isobaric diffusion flux in a ternary mixture with Fick’s law requires a matrix with four different diffusion coefficient elements which depend not only on the composition, but also on the velocity reference frame and the choice of the component order. It has been shown that a change of the reference frame for mixtures with large excess volume may even lead to seemingly negative main Fick diffusion matrix elements^14^. On the other hand, most predictive equations for transport diffusion coefficients of multicomponent liquids are based on the Darken relation^15–17^ which neglects cross-effects. Therefore, they are not valid for mixtures with strong intermolecular interactions and are thus not useful for many practical applications. Further, the development and validation of empirical correlations and theory-based predictive equations is hindered by the lack of experimental data.

In contrast to the rather slow progress of experimental work, the capabilities of molecular dynamics simulation techniques to investigate diffusion processes have experienced a rapid development, driven by the increase of computational power following Moore’s law and the continuous improvement of specific algorithms. Half a century after the pioneering work of Alder and Wainwright^18^ on the velocity auto-correlation function of hard spheres, molecular dynamics simulation has become a powerful method to understand, model and predict diffusion processes in scientific and engineering applications. Nowadays, it is not only able to deal with more complex force fields and much larger systems, but it also reaches longer time scales, which allows for accurate predictions of the Fick diffusion coefficient of highly non-ideal multicomponent mixtures^12^. Molecular simulation is well-suited to provide trustworthy diffusion coefficient data and may contribute to the understanding of the underlying microscopic processes that are not accessible with experiments.

Recently, Fick diffusion coefficient data for a quaternary real mixture calculated solely with molecular dynamics simulation techniques were reported for the first time^12^. The regarded mixture water + methanol + ethanol + 2-propanol exhibits strong intermolecular interactions mainly due to hydrogen bonding. Molecular dynamics simulations of such mixtures are challenging because they require a large number of molecules to be sampled over a relatively long time interval to obtain acceptable statistical uncertainties. In this work, a close look at the quaternary Fick diffusion matrix is given in the light of varying velocity reference frames and component orders. Issues related to the finite size correction of the sampled diffusion coefficients are discussed as well. Further, Fick diffusion coefficient data for the ternary subsystems water + methanol + 2-propanol, water + ethanol + 2-propanol and methanol + ethanol + 2-propanol obtained by molecular simulation are presented here for the first time. The capability of the force fields and the methodology employed in this work to accurately predict Fick diffusion coefficients of most binary^19,20^ and one ternary subsystem^21,22^ has already been demonstrated. Therefore, the present data are expected to be realistic.

## Results

The most common approach to address mass transport in liquid mixtures is Fick’s law, which describes the molar flux in a mixture by means of a linear combination of the mole fraction gradients. However, chemical potential gradients and not mole fraction gradients are the true thermodynamic driving forces for diffusion. Maxwell–Stefan theory follows this path, assuming that chemical potential gradients are balanced by friction forces between the components. Maxwell–Stefan diffusion coefficients cannot be measured in the laboratory, but are directly accessible with equilibrium molecular dynamics. On the other hand, Fick diffusion coefficients are obtained from experiments. Because the Fick and Maxwell–Stefan approaches describe the same process, there is a straightforward relation between the according coefficients given by the so-called thermodynamic factor. In this work, equilibrium molecular dynamics simulation and the Green–Kubo formalism were employed to obtain the Maxwell–Stefan diffusion coefficient and Kirkwood–Buff integration^23,24^ to sample the thermodynamic factor consistently on the basis of the chosen force field model.

**Figure 1:**
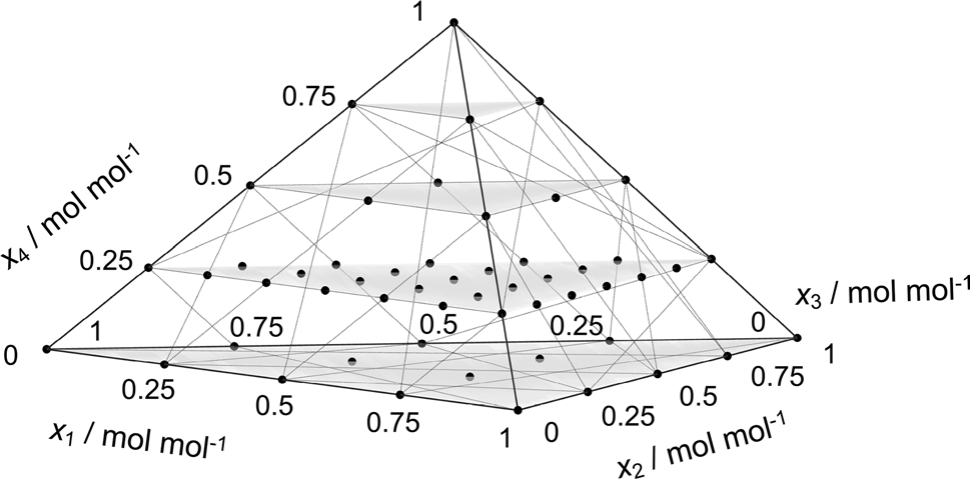
Studied compositions (bullets) of the quaternary mixture water (1) + methanol (2) + ethanol (3) + 2-propanol (4) as well as its ternary and binary subsystems.

The Fick diffusion coefficient in the molar reference frame of the quaternary mixture water + methanol + ethanol + 2-propanol was reported in previous work^12^ for ten compositions along the isopleth $x_{\mathrm{C3H8O}} = 0.25$ $\hbox {mol}\,\hbox {mol}^{-1}$. Here, these data are discussed together with new simulation results for the thermodynamic factor, Fick diffusion coefficient and shear viscosity of all four ternary and six binary subsystems at 298.15 K and 0.1 MPa. The studied state points are depicted in Fig. 1. The ternary subsystem water + methanol + ethanol is discussed in less detail because it was addressed in Refs.^21,22^.

### Thermodynamic factor

The thermodynamic factor $\Gamma _{ij}$ describes the thermodynamic non-ideality of a mixture. It can be estimated from experimental vapor-liquid equilibrium or excess enthalpy data^25,26^, employing an equation of state or an excess Gibbs energy model because it is related to the composition dependence of the chemical potentials by^27^

1 $$\begin{aligned} \Gamma _{ij}= \frac{x_i}{k_\text{B}T}\frac{\partial \mu _i}{\partial x_j}\bigg |_{T,p,x_{k,k\ne j = 1 \dots n-1}}=\delta _{ij}+x_i\frac{\partial \ln \gamma _i}{\partial x_j}\bigg |_{T,p,x_{k,k\ne j = 1 \dots n-1}}, \end{aligned}$$

where $\delta _{ij}$ is the Kronecker delta, $k_\text{B}$ the Boltzmann constant and *T* the temperature. $x_i$, $\mu _i$ and $\gamma _i$ are the mole fraction, chemical potential and activity coefficient of component *i*, respectively. The differential has to be carried out keeping the mole fraction of all other components $x_{k, k\ne j}$ constant, except for the *n*th. The mole fraction of component *n* is eliminated by the fact that the mole fractions sum up to unity. However, the thermodynamic factor can be also sampled directly by molecular simulation, e.g. with Kirkwood–Buff integration^24,28^ or free energy perturbation methods, to obtain the composition dependence of the chemical potential^21,29^. For the sake of consistency, the thermodynamic factor was calculated here with Kirkwood–Buff integration as described in preceding work^12,23^.

**Figure 2:**
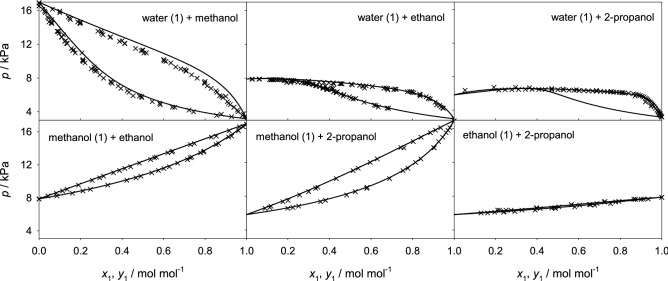
Vapor-liquid equilibria of the six binary subsystems at 298.15 K. Simulation-based Wilson predictions (lines) are compared with experimental literature data^30–38^ (crosses).

**Figure 3:**
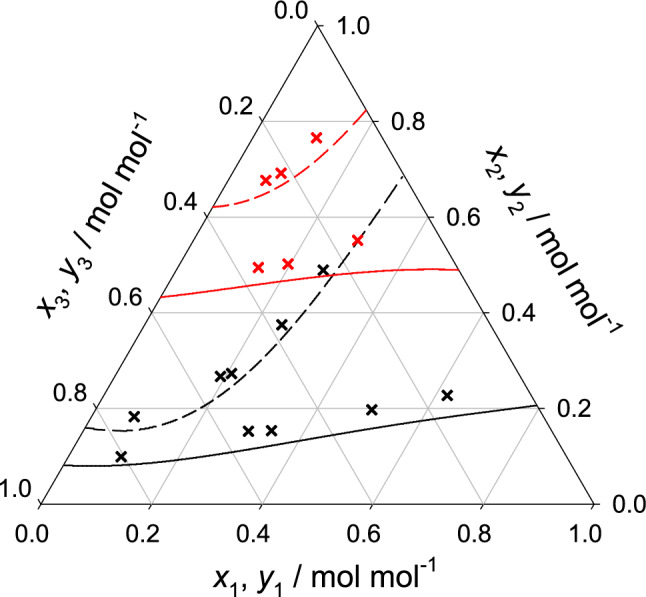
Vapor-liquid equilibria of the ternary subsystem water (1) + methanol (2) + ethanol (3) at 298.15 K and 11.819 kPa (red) as well as 8.628 kPa (black). Simulation-based Wilson predictions of the saturated liquid composition (line) and saturated vapor composition (dashed line) are compared with experimental literature data^39^ (crosses).

In order to assess the reliability of the sampled thermodynamic factor, vapor-liquid equilibrium data were predicted with the Wilson excess Gibbs energy model that was solely fitted to the thermodynamic factor results for all studied binary and ternary mixtures and compared with experimental literature data wherever available. Figure 2 shows the resulting vapor-liquid equilibrium data for all six binary subsystems. In general, an excellent agreement was found, merely for water + methanol there is a small overestimation of the vapor pressure, which can be explained by the limitations of the force field model in absence of adjustable binary parameters. In the case of the ternary subsystems, a relatively good agreement was found between simulation and experiment for water + methanol + ethanol. The small discrepancies observed for this ternary mixture can be traced back to the vapor pressure shift observed for its binary subsystem water + methanol. Further comparisons were not possible because of the lack of experimental data at the studied thermodynamic conditions, cf. Fig. 3.

### Fick diffusion coefficient

The Fick diffusion coefficient matrix in the molar reference frame $\mathbf {D}^{\mathrm{M}}$ of mixtures with *n* components was calculated from the phenomenological diffusion coefficients $L_{ij}$ and the thermodynamic factor matrix ${\varvec{\Gamma }}$ sampled directly with molecular simulation, employing the relation $[\mathbf {D}^{\mathrm{M}}]=[\mathbf {B}]^{-1} [{\varvec{\Gamma }}],$ in which all three symbols represent $(n-1)\times (n-1)$ matrices and $[{\varvec{B}}]=[{\varvec{\Delta }}]^{-1}$, where

2 $$\begin{aligned} \Delta _{ij}= (1-x_i)\left( \frac{L_{ij}}{x_j}-\frac{L_{in}}{x_n}\right) -x_i\sum ^{n}_{k=1\ne i}\left( \frac{L_{kj}}{x_j}-\frac{L_{kn}}{x_n}\right) . \end{aligned}$$

**Figure 4:**
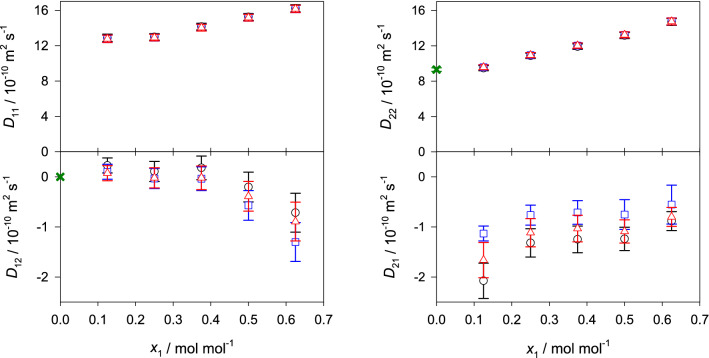
Elements of the Fick diffusion coefficient matrix in the molar (black circles), volume (blue squares) and mass reference frame (red triangles) of the ternary subsystem methanol (1) + ethanol (2) + 2-propanol (3) with $x_{3}=0.25$ $\hbox {mol}\,\hbox {mol}^{-1}$ at 298.15 K and 0.1 MPa. The green crosses represent the expected values in the binary limit $x_1 \rightarrow 0$ for the molar reference frame.

**Figure 5:**
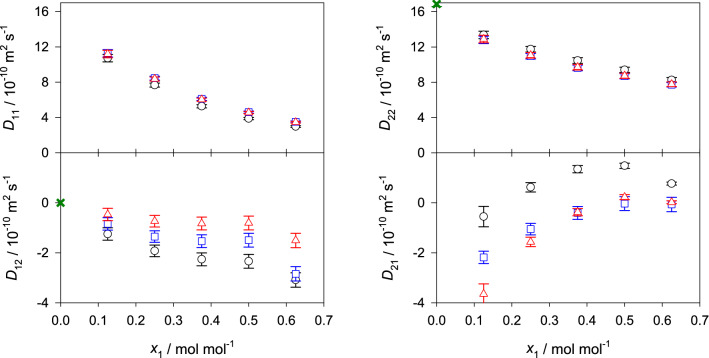
Elements of the Fick diffusion coefficient matrix in the molar (black circles), volume (blue squares) and mass reference frame (red triangles) of the ternary subsystem water (1) + methanol (2) + 2-propanol (3) with $x_{3}=0.25$ $\hbox {mol}\,\hbox {mol}^{-1}$ at 298.15 K and 0.1 MPa. The green crosses represent the expected values in the binary limit $x_1 \rightarrow 0$ for the molar reference frame.

**Figure 6:**
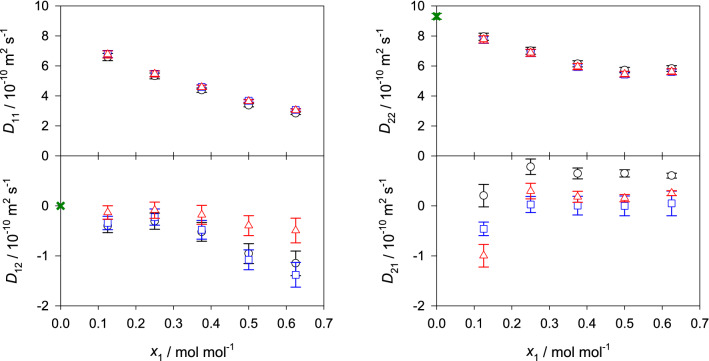
Elements of the Fick diffusion coefficient matrix in the molar (black circles), volume (blue squares) and mass reference frame (red triangles) of the ternary subsystem water (1) + ethanol (2) + 2-propanol (3) with $x_{3}=0.25$ $\hbox {mol}\,\hbox {mol}^{-1}$ at 298.15 K and 0.1 MPa. The green crosses represent the expected values in the binary limit $x_1 \rightarrow 0$ for the molar reference frame.

The corresponding equations for the binary mixtures are given in the supplementary section II online. Figures 4, 5 and 6 show the predicted values of the Fick diffusion coefficient matrix of the three ternary subsystems in different reference frames when the mole fraction of 2-propanol is kept constant, i.e. $x_{\mathrm{C3H8O}} = 0.25$ $\hbox {mol}\,\hbox {mol}^{-1}$. The predictions for the fourth ternary subsystem water + methanol + ethanol are presented in the supplementary Fig. S1 online. In case of the ternary subsystem consisting of alcohols only, both main elements of the Fick diffusion coefficient matrix $D_{ii}$ generally increase with rising methanol concentration, which can be explained by the difference in size of methanol and ethanol molecules, i.e. smaller methanol molecules replace larger ethanol molecules, leading to faster diffusion. Both cross elements $D_{ij}$ increase in magnitude with rising methanol mole fraction, which indicates stronger association between methanol and ethanol molecules.

**Figure 7:**
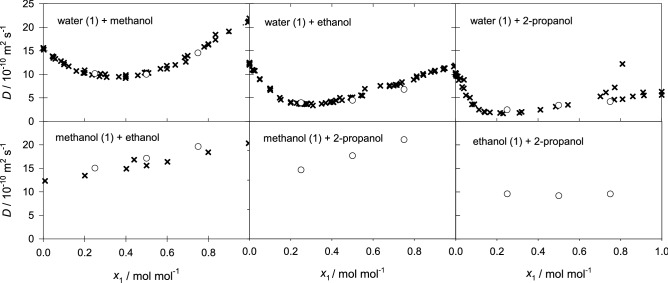
Fick diffusion coefficient of the six binary subsystems at 298.15 K and 0.1 MPa. Present simulation results (circles) are compared with experimental literature data^21,40–48^ (crosses). The error bars are within symbol size.

In case of the aqueous ternary subsystems, both main elements of the Fick diffusion coefficient matrix decrease with rising water mole fraction, which indicates an expansion of the hydrogen-bonding network and clustering that hinder diffusion. Generally, the cross element $D_{12}$ increases in magnitude with rising water mole fraction, while $D_{21}$ decreases. Unfortunately, there are no experimental Fick diffusion coefficient data for the studied ternary mixtures to assess the present predicted values. However, a good agreement with experimental values is expected because of the successful prediction of the Fick diffusion coefficient of most of the binary subsystems and the ternary subsystem water + methanol + ethanol, cf. Fig. 7 and Ref.^22^. Further, the predicted values are consistent with the expected asymptotic behavior in the limit of vanishing water concentration. This type of analysis has been outlined in previous work^12,22^ and is briefly described in the supplementary material section II online. The corresponding numerical data are given in the supplementary Tables S1 to S3 online.

#### Influence of reference frame

In molecular dynamics simulations, the molar reference frame is usually employed to obtain Fick diffusion coefficients. On the other hand, experimental data are typically evaluated in the volume reference frame. To compare among different approaches, it is important to be aware how the according Fick diffusion coefficients relate to each other. The diffusion coefficient matrix in the molar reference frame $\mathbf {D}^{M}$ can be transformed into its form in the volume reference frame $\mathbf {D}^{V}$ employing $[ \mathbf {D}^{V} ] = [\mathbf {B}^{Vu}][\mathbf {D}^{M}][\mathbf {B}^{uV}$] with

3 $$\begin{aligned} B^{Vu}_{ik} = \delta _{ik} - x_i\left( v_{k}-v_{n}\right) / v, \nonumber \\ B^{uV}_{ik} = \delta _{ik} - x_i\left( 1- v_{k}/v_{n}\right) , \end{aligned}$$

where $B^{Vu}_{ik}$ and $B^{uV}_{ik}$ are the elements of the $\mathbf {B}^{Vu}$ and $\mathbf {B}^{uV}$ matrices, respectively. *v* is the total molar volume $v =\sum _{i=1}^{n} x_i v_{i}$ and the *n* indicates the reference component. The required partial molar volumes $v_i$ were calculated from the composition dependence of the total molar volume of the mixture obtained from a fit of experimental data^49^. The corresponding equations for the transformation between the reference frames are given in the supplementary material section II online.

**Figure 8:**
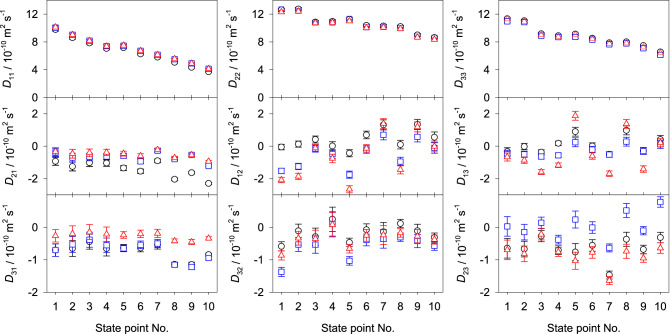
Elements of the Fick diffusion coefficient matrix in the molar (black circles), volume (blue squares) and mass reference frame (red triangles) of the quaternary mixture water (1) + methanol (2) + ethanol (3) + 2-propanol (4) for the regarded ten state points at 298.15 K and 0.1 MPa. The molar composition of the regarded state points is given in the supplementary Table S4 online.

Here, the influence of varying velocity reference frame on the elements of the Fick diffusion coefficient matrix was studied for the regarded ternary and quaternary mixtures, cf. Figs. 4, 5, 6 and 8 . Generally, the main elements are less sensitive to the reference frame than the cross elements of the diffusion matrix. However, for the regarded mixtures, the values of the main elements change between 0.1 and 19% when transformed into the volume or mass reference frame. On the other hand, the cross elements may change by more than one order of magnitude. Consequently, cross elements that could be considered as negligible in the molar reference frame are not negligible in the volume or mass reference frame or vice versa. This evidences that the interpretation of the Fick diffusion coefficient matrix has to be done carefully. Thus, the potential presence of uphill diffusion and serpentine composition trajectories^13^ due to significant cross-effects cannot be analyzed solely on the basis of the cross elements of the Fick diffusion coefficient matrix in one distinct reference frame. It should be noted that the smallest influence of the reference frame on the diffusion matrix was found for the ternary mixture containing only alcohols, which can be explained by their molecular similarity.

#### Influence of component order

There are different ways to order the components in a multicomponent mixture. Usually, the species with the highest concentration is chosen as the “solvent” due to accuracy concerns. However, the choice of component order is arbitrary and although a varying component order changes the values of the Fick diffusion matrix, the actual fluxes remain unchanged. Therefore, the elements of the Fick diffusion matrix for a specific component order can be rewritten as a linear combination of the elements of the Fick diffusion matrix for any other component order. The equations to transform the Fick diffusion coefficient matrix for different component orders are given in the supplementary material section II online.

**Figure 9:**
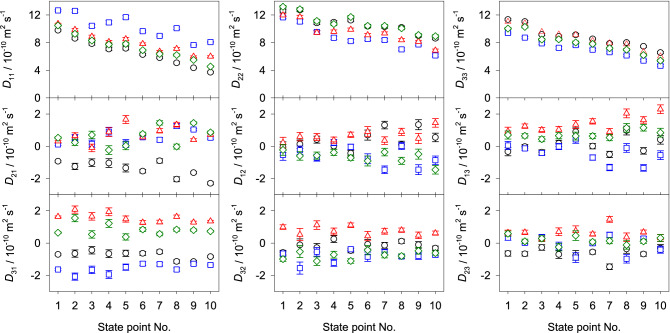
Elements of the Fick diffusion coefficient matrix in the molar velocity reference frame of the quaternary mixture water (W) + methanol (M) + ethanol (E) + 2-propanol (P) with varying component order: MEPW (blue squares), WEPM (red triangles), WMPE (green diamonds) and WMEP (black circles) at 298.15 K and 0.1 MPa. The last component is regarded as the solvent. Note that the indices *i* and *j* of $D_{ij}$ refer to the elements of the diffusion matrix for the corresponding component order. The molar composition of these state points is given in the supplementary Table S4 online.

Figure 9 shows how the elements of the Fick diffusion coefficient matrix in the molar reference frame vary when the solvent is changed. The main element $D_{11}$ increases with falling molar mass of the solvent, i.e. the highest values are those for water as the solvent. On the other hand, the highest values for $D_{22}$ are found for ethanol and 2-propanol as solvents. Further, values of the elements $D_{22}$ and $D_{33}$ are the lowest when water is the solvent. Cross elements are more sensitive to the component order. They show a larger change in magnitude than the main elements when the solvent is varied, in several cases they even exhibit a change in sign. Numerical data are given in the supplementary Table S5 online.

### Shear viscosity

**Figure 10:**
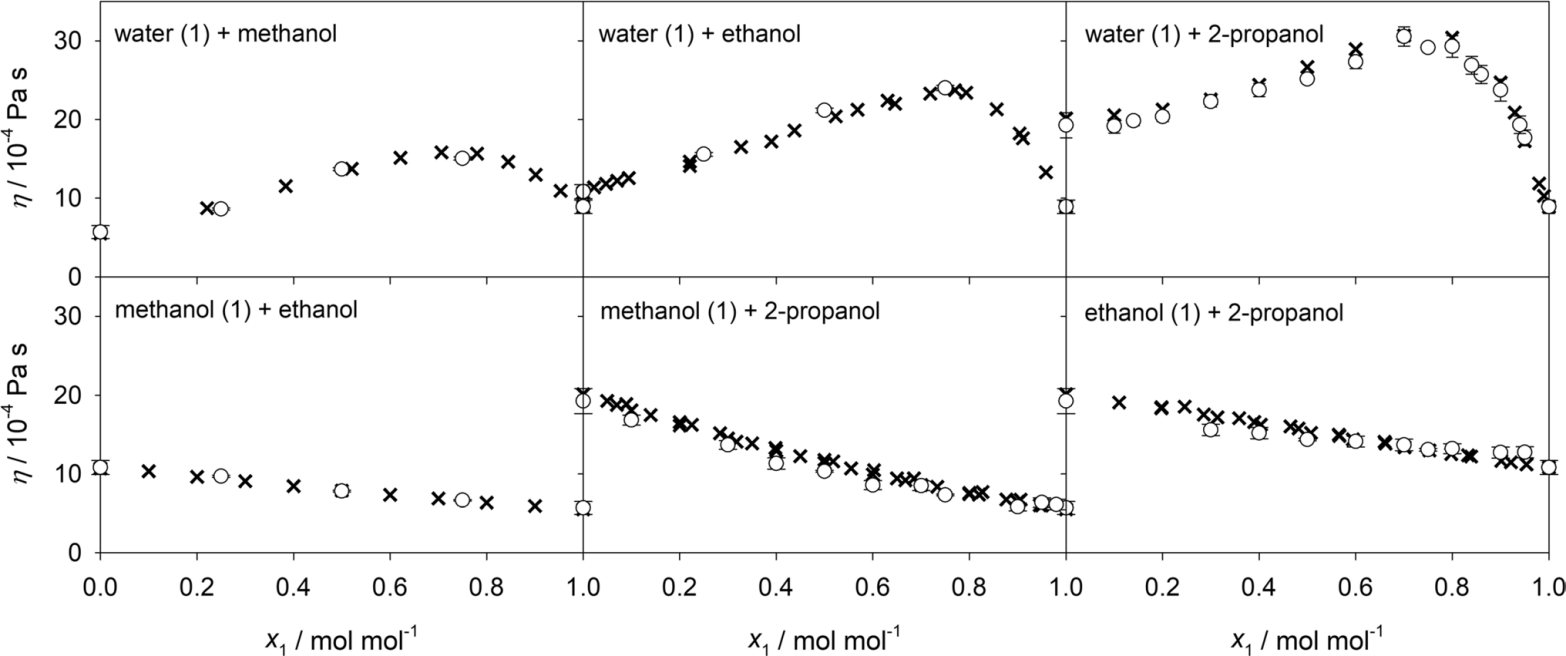
Shear viscosity of the six binary subsystems at 298.15 K and 0.1 MPa. Present simulation results (circles) are compared with experimental literature data^52–55^ (crosses).

**Figure 11:**
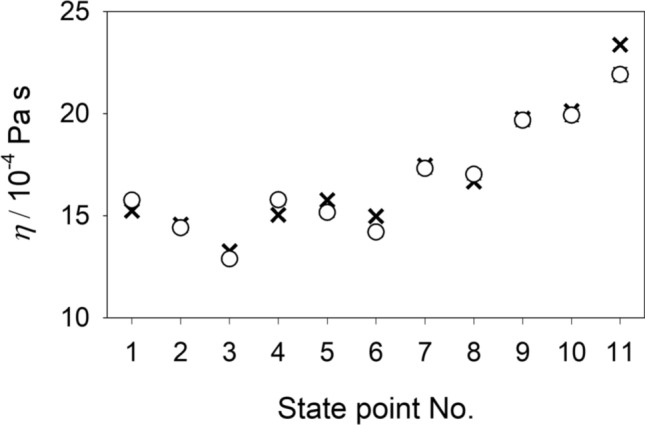
Shear viscosity of the quaternary mixture water + methanol + ethanol + 2-propanol at 298.15 K and 0.1 MPa. The McAllister^51^ equation fit to present molecular simulation results (circles) is compared with experimental literature data^56^ (crosses). The molar composition of these state points is given in the supplementary Table S7 online.

Viscosity is closely related to diffusion as exploited e.g. by the Stokes–Einstein equation^50^. Thus, an adequate prediction of the shear viscosity suggests the reliability of the predicted diffusion coefficients. Because of the lack of experimental data on transport diffusion coefficients of multicomponent mixtures, it is compelling to consider experimental shear viscosity data. Despite the strong viscous non-idealities of aqueous alcoholic mixtures, molecular modeling and simulation is able to predict the composition dependence of the shear viscosity with an absolute average deviation of 5.4% for all binary subsystems, cf. Fig. 10. In order to compare the predicted shear viscosity with experimental literature data for the ternary and quaternary mixtures, the McAllister four body equation^51^ was fitted to the present molecular simulation results. As can be seen in Fig. 11, the resulting fit agrees well with the available experimental values with an average relative deviation of 1.8%. The absolute average deviation for all ternary mixtures is 7.5%. Numerical data are given in the supplementary Table S6 online.

## Discussion and conclusion

A comprehensive study on the Fick diffusion coefficient matrix of the quaternary mixture water + methanol + ethanol + 2-propanol and its subsystems was conducted at 298.15 K and 0.1 MPa. The Maxwell–Stefan diffusion coefficient was sampled with equilibrium molecular dynamics and the Green–Kubo formalism, employing rigid, non-polarizable force fields based on Lennard–Jones sites and superimposed point charges. A thorough analysis of system size effects and the corresponding corrections was carried out. The thermodynamic factor was calculated via Kirkwood–Buff integration. In this way, the Fick diffusion coefficient matrix was determined consistently on the basis of the selected force field model. The predictive power of the employed molecular simulation techniques was confirmed by a satisfactory comparison with experimental vapor-liquid equilibrium and Fick diffusion data of the binary subsystems. Additional confidence about the present results is provided by the good agreement between the predicted shear viscosity and experimental literature data for the ternary and quaternary mixtures^56^.

The influence of the reference frame on the Fick diffusion coefficient matrix of the quaternary mixture and its ternary subsystems was also analyzed. It was found that the main elements of the diffusion matrix exhibit a weaker dependence on the reference frame than the cross elements. As expected, the ternary mixture consisting solely of alcohols shows smaller variations among different reference frames. On the other hand, the main elements of the diffusion matrix of the aqueous mixtures may differ by up to 19% when the reference frame is varied. This can in part be explained by the relatively large differences between the partial molar volume of water and its pure substance volume, which may reach up to $-17$%^49^. The substantial dependence of the cross elements of the Fick diffusion coefficient matrix on the reference frame makes its interpretation challenging. Further, it also depends on the component order, which was shown to lead to strong variations of the cross elements for the studied mixtures. Thus, the presence of strong coupling effects related to uphill diffusion phenomena cannot be directly inferred from the ratio between main and cross elements of any given Fick diffusion coefficient matrix. Clearly, the physical interpretation of this matrix deserves future investigations, e.g. with an extensive analysis of frame-independent Fick diffusion coefficients as proposed by Ortiz de Zárate and Sengers^57^.

## Methods

Equilibrium molecular dynamics simulations of the quaternary mixture water + methanol + ethanol + 2-propanol as well as all of its ternary and binary subsystems were performed, employing rigid, non-polarizable force fields based on Lennard–Jones sites and superimposed point charges that may or may not coincide with their site positions^20,58–60^. A detailed description of these force fields is given in the supplementary material online. Their capability to adequately predict the self-diffusion coefficient and the shear viscosity of the pure fluids and the aqueous alcoholic binary mixtures has been reported in previous work^20,61,62^. Further, these models were successfully employed to predict the Fick diffusion coefficient matrix of the ternary mixture water + methanol + ethanol^21,22^.

Transport data were sampled by equilibrium molecular dynamics and the Green–Kubo formalism. This approach was preferred over non-equilibrium methods because intra- and transport diffusion coefficients as well as shear viscosity can be sampled concurrently. For an arbitrary transport coefficient $\Xi$, the generic Green–Kubo expression is

4 $$\begin{aligned} \Xi =\frac{1}{G}\int _0^{\infty } dt~\big \langle  {\dot{A}}(t)\cdot {{\dot{A}}}(0)\big \rangle , \end{aligned}$$

where *G* is a transport property specific pre-factor, $\mathbf {A}$ the related perturbation, ${{\dot{A}}}$ its time derivative and the brackets $<\cdots>$ denote ensemble averaging. The working equations for the sampling of the intra-diffusion and phenomenological coefficients as well as the shear viscosity are given in the supplementary material section III online together with technical simulation details.

The thermodynamic factor of the studied mixtures was estimated from the microscopic structure based on Kirkwood–Buff integrals $G_{ij}$^63^

5 $$\begin{aligned} G_{ij} = 4 \pi \int _{0}^{\infty } \left( g_{ij}(r)-1\right) r^2dr, \end{aligned}$$

where $g_{ij}\,(r)$ is the radial distribution function. Because Eq. (5) is defined for the grand canonical ensemble, convergence issues have to be expected when the canonical ensemble is employed^64^ so that corrections are required. For this purpose, the truncation method by Krüger et al.^65^ was applied here. Moreover, corrections of the radial distribution function based on the method by Ganguly and van der Vegt^66^ were employed. Extrapolation to the thermodynamic limit was not necessary in this context because of the rather large ensembles containing $N = 6000$ molecules. Expressions for the sampling of the thermodynamic factor matrix have been derived by Fingerhut et al.^23^ based on Ben-Naim’s formalism to determine mole number derivatives of the chemical potential from Kirkwood–Buff integrals^67^ and are not repeated here.

### Finite size corrections

It is well known that small molecular systems under periodic boundary conditions are associated with systematic errors when diffusion coefficients are calculated. The correction by Yeh and Hummer^68^ (YH) is the most widely employed method to account for finite size effects. This correction, based on the shear viscosity $\eta$ and the edge length of the simulation volume *L*, i.e. $2.837297\cdot k_B T / (6\pi \eta L)$, is widely used since it does not require additional simulation runs. However, it has been demonstrated that the YH correction term is not always adequate^69^. A review on this topic was recently published by Celebi et al.^70^.

For multicomponent mixtures, an approach based on the correction of the underlying phenomenological coefficients $L_{ij}$ instead of Maxwell–Stefan or Fick diffusion coefficients^71^ was proposed in previous work^12^. Because the present quaternary mixture is dominated by strong intermolecular interactions, a significant system size dependence was only found for the main phenomenological coefficients $L_{ii}$, whereas no clear system size dependence was identified for the strongly scattering cross phenomenological coefficients $L_{ij}$. Here, the system size dependence of three ternary mixtures was studied by performing series of simulations with varying system size containing 512–8000 molecules. The infinite size values were obtained from the extrapolation $1/L \rightarrow 0$ of the intra- and phenomenological diffusion coefficients.

In case of the intra-diffusion coefficients, the YH correction term yields an overestimation between 10% and 4% for systems containing 1000 and 8000 molecules, respectively, cf. supplementary Figs. S2, S3, S4 and S5 online. Note that the statistical uncertainty of the simulation results was throughout below 0.5%.

**Figure 12:**
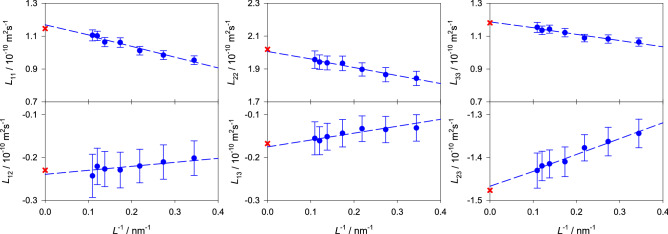
Phenomenological coefficients of the ternary subsystem water (1) + ethanol (2) + 2-propanol (3) ($x_{\mathrm{H2O}}=0.125$, $x_{\mathrm{C2H6O}}=0.625$ and $x_{\mathrm{C3H8O}}=0.25$ $\hbox {mol}\,\hbox {mol}^{-1}$) as a function of the inverse edge length of the simulation volume *L* at 298.15 K and 0.1 MPa. The uncorrected simulation results (blue bullets) are shown together with the corrected values using the fast correction procedure^12^ for $N= 6000$ (red crosses). The blue dashed line is a linear fit to the uncorrected simulation results.

Similarly, the values for an infinite system size were calculated for all main $L_{ii}$ and cross phenomenological coefficients $L_{ij}$ of the studied ternary mixtures. The fast correction procedure based on normalized coefficient values^12^ leads to corrections of the main and cross phenomenological coefficients for simulations with 6000 molecules of approximately 5% and 4%, respectively. Infinite size extrapolated and corrected diffusion values exhibit a good agreement, with relative deviations below 1.5% and 4%, respectively. A graphical comparison is shown in Fig. 12 for the mixture water + ethanol + 2-propanol. Further examples can be found in the supplementary Figs. S6, S7 and S8 online.

Because the phenomenological coefficients are underlying to the Maxwell–Stefan and Fick diffusion coefficients, both are expected to be associated with finite size effects. The extrapolated values for infinite size agree on average within 1.8% with the ones based on the fast correction procedure for 6000 molecules. Graphical comparisons are shown in the supplementary Figs. S9, S10, S11 and S12 online.

The Fick diffusion coefficient matrix is indeed associated with finite size effects. A strong size dependence was observed for both main elements, whereas a weaker size dependence was found for the cross elements that was in many cases difficult to identify due to data scattering. Jamali et al.^71^ proposed a YH-based correction only for the main elements of the Fick diffusion matrix and did not identify any size dependence of the cross elements in their data, which can be explained by data scattering covering up weak size effects. In fact, finite size effects observed for the Fick diffusion matrix are the logical consequence of the ones of the underlying phenomenological coefficients. When data scattering is reduced, e.g. by employing a linear fit to the phenomenological coefficients, a clear system size dependence is observed even for the cross elements where it could not be recognized otherwise, cf. supplementary Fig. S13 online.

**Figure 13:**
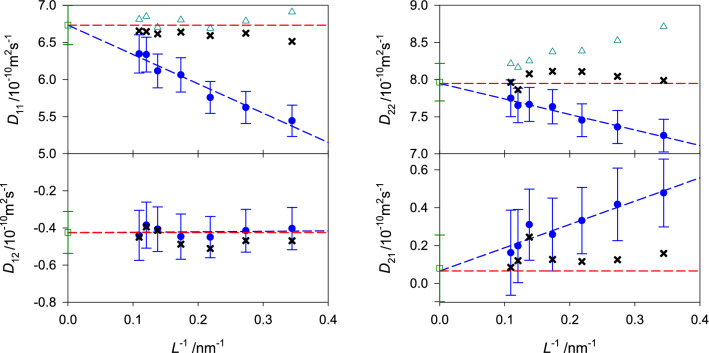
Elements of the Fick diffusion coefficient matrix of the ternary subsystem water (1) + ethanol (2) + 2-propanol (3) ($x_{\mathrm{H2O}}=0.125$, $x_{\mathrm{C2H6O}}=0.625$ and $x_{\mathrm{C3H8O}}=0.25$ $\hbox {mol}\,\hbox {mol}^{-1}$) as a function of the inverse edge length of the simulation volume *L* at 298.15 K and 0.1 MPa. The blue dashed line is a linear fit to the uncorrected simulation results (blue bullets). The coefficients calculated with the corrected values using the fast correction procedure^12^ (crosses) are compared with those according to the procedure by Jamali et al.^71^ (cyan triangles). The green squares represent the Fick diffusion coefficient values based on the individually extrapolated phenomenological coefficients.

A comparison was made between the YH correction by Jamali et al.^71^ and the fast correction method based on the phenomenological coefficient^12^. For the main Fick diffusion coefficient element $D_{11}$, both approaches usually yield an excellent agreement with the extrapolated infinite size value for systems with more than $1000$ molecules. In case of $D_{22}$, the magnitude of the size effect is clearly overestimated by the YH term. For the mixtures studied in this work, it is evident that the required system size correction is different for each of the main elements of the diffusion matrix. However, the correction method by Jamali et al.^71^ corrects both main elements by the same value, which can explain the overestimation of $D_{22}$. For the cross Fick diffusion coefficient elements, the fast correction method is usually in good agreement with the extrapolated values, especially when a strong size dependence is present, cf. Fig. 13 and supplementary Figs. S14, S15 and S16 online. Therefore, the Fick diffusion coefficient data from molecular simulation were calculated here throughout with the fast correction of all phenomenological coefficients^12^.

## Supplementary Information

Supplementary Information 1.

## Data Availability

All data analyzed in this study are included in this published article and its Supplementary Material files. Other data are available from the corresponding author upon request.
