# Supplementary material for: Diffusion in multicomponent aqueous alcoholic mixtures
Source: Sci Rep. 2021 Jun 10;11:12319. doi: 10.1038/s41598-021-91727-w (PMC8192923; doi:10.1038/s41598-021-91727-w)
Supplement: Supplementary file 1 — Supplementary Information 1. [file 41598_2021_91727_MOESM1_ESM.pdf]

Supplementary material to:
Diffusion in multicomponent aqueous alcoholic mixtures

Gabriela Guevara-Carrion, Robin Fingerhut, and Jadran Vrabec*
Thermodynamics and Process Engineering, Technische Universität Berlin, Ernst-Reuter-Platz 1, 10587 Berlin, Germany
(Dated: June 2, 2021)

CONTENTS

I. Simulation results 2
Fick diffusion coefficient matrix 2
Numerical results 3

II. Fick diffusion coefficient matrix 7
Asymptotic behavior of the ternary Fick diffusion matrix 7
Frame of reference 7
Choice of the solvent 8

III. Molecular Simulation 9
Force fields 9
Phenomenological coefficients 10
Shear viscosity 11
Technical details 11

IV. Finite size effects 11
Intra-diffusion coefficients 12
Phenomenological coefficients 14
Maxwell-Stefan diffusion coefficients 17
Fick diffusion coefficient 19

References 23

* vrabec@tu-berlin.de

I. SIMULATION RESULTS

Fick diffusion coefficient matrix

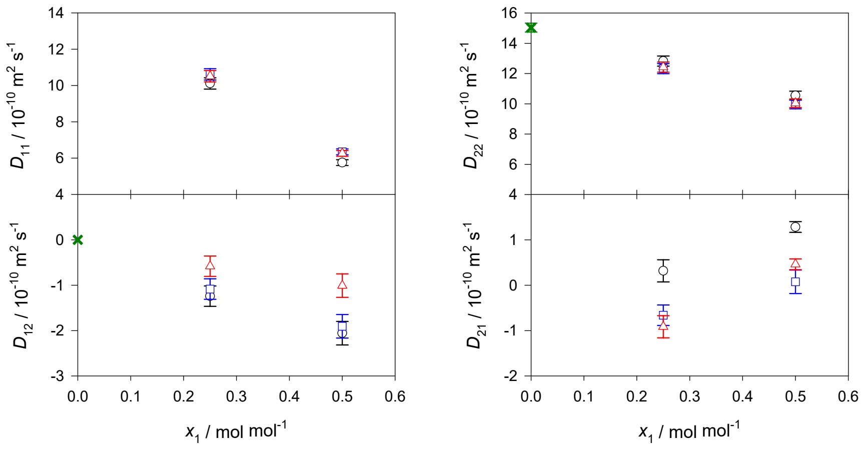

FIG. S1. Elements of the Fick diffusion coefficient matrix in the molar (black circles), volume (blue squares) and mass reference frame (red triangles) of the ternary subsystem water (1) + methanol (2) + ethanol (3) with $x_3 = 0.25$ mol·mol^{-1} at 298.15 K and 0.1 MPa. The green crosses represent the expected value in the binary limit $x_1 \to 0$ for the molar reference frame.

Numerical results

TABLE S1. Fick diffusion coefficient matrix in the molar, volume and mass reference frame of the ternary subsystem methanol (1) + ethanol (2) + 2-propanol (3) at 298.15 K and 0.1 MPa. The numbers in parentheses indicate the uncertainty in the last given digit.

No.	x_1	x_2	$\mathbf{D}^M$	$\mathbf{D}^V$	$\mathbf{D}^w$
	mol·mol^{-1}		10^{-10}m^{2}s^{-1}	10^{-10}m^{2}s^{-1}	10^{-10}m^{2}s^{-1}
1	0.125	0.625	$\begin{pmatrix} 12.9(3) & 0.2(1) \\ -2.1(3) & 9.5(3) \end{pmatrix}$	$\begin{pmatrix} 12.8(3) & 0.1(1) \\ -1.1(2) & 9.6(3) \end{pmatrix}$	$\begin{pmatrix} 12.8(3) & 0.1(1) \\ -1.7(2) & 9.6(3) \end{pmatrix}$
2	0.250	0.500	$\begin{pmatrix} 13.0(3) & 0.1(2) \\ -1.3(3) & 10.9(3) \end{pmatrix}$	$\begin{pmatrix} 12.9(3) & -0.0(2) \\ -0.8(2) & 10.9(3) \end{pmatrix}$	$\begin{pmatrix} 12.9(3) & -0.0(2) \\ -1.1(2) & 10.9(3) \end{pmatrix}$
3	0.375	0.375	$\begin{pmatrix} 14.2(4) & 0.2(2) \\ -1.2(3) & 11.9(3) \end{pmatrix}$	$\begin{pmatrix} 14.0(4) & -0.0(2) \\ -0.7(3) & 12.0(3) \end{pmatrix}$	$\begin{pmatrix} 14.0(4) & -0.0(2) \\ -1.0(3) & 12.0(3) \end{pmatrix}$
4	0.500	0.250	$\begin{pmatrix} 15.3(4) & -0.2(2) \\ -1.2(2) & 13.2(3) \end{pmatrix}$	$\begin{pmatrix} 15.2(4) & -0.6(2) \\ -0.8(3) & 13.2(3) \end{pmatrix}$	$\begin{pmatrix} 15.2(4) & -0.4(2) \\ -1.1(3) & 13.2(3) \end{pmatrix}$
5	0.625	0.125	$\begin{pmatrix} 16.2(4) & -0.7(3) \\ -0.9(2) & 14.7(4) \end{pmatrix}$	$\begin{pmatrix} 16.2(4) & -1.3(3) \\ -0.6(3) & 14.8(4) \end{pmatrix}$	$\begin{pmatrix} 16.2(4) & -0.9(3) \\ -0.8(3) & 14.8(4) \end{pmatrix}$
6	0.250	0.250	$\begin{pmatrix} 11.9(3) & -0.1(2) \\ -0.6(2) & 10.7(3) \end{pmatrix}$	$\begin{pmatrix} 11.9(3) & -0.2(2) \\ -0.4(2) & 10.7(3) \end{pmatrix}$	$\begin{pmatrix} 11.9(3) & -0.2(2) \\ -0.5(2) & 10.7(3) \end{pmatrix}$

TABLE S2. Fick diffusion coefficient matrix in the molar, volume and mass reference frame of the ternary subsystem water (1) + methanol (2) + 2-propanol (3) at 298.15 K and 0.1 MPa. The numbers in parentheses indicate the uncertainty in the last given digit.

No.	x_1	x_2	$\mathbf{D}^M$	$\mathbf{D}^V$	$\mathbf{D}^w$
	mol·mol^{-1}		10^{-10}m^{2}s^{-1}	10^{-10}m^{2}s^{-1}	10^{-10}m^{2}s^{-1}
1	0.125	0.625	$\begin{pmatrix} 10.7(4) & -1.2(2) \\ -0.6(3) & 13.4(4) \end{pmatrix}$	$\begin{pmatrix} 11.3(4) & -0.8(2) \\ -2.2(3) & 12.8(4) \end{pmatrix}$	$\begin{pmatrix} 11.2(4) & -0.5(2) \\ -3.6(3) & 12.9(4) \end{pmatrix}$
2	0.250	0.500	$\begin{pmatrix} 7.7(2) & -1.9(2) \\ 0.6(2) & 11.7(3) \end{pmatrix}$	$\begin{pmatrix} 8.5(2) & -1.3(2) \\ -1.1(2) & 11.0(3) \end{pmatrix}$	$\begin{pmatrix} 8.4(2) & -0.7(2) \\ -1.6(2) & 11.1(3) \end{pmatrix}$
3	0.375	0.375	$\begin{pmatrix} 5.3(2) & -2.3(2) \\ 1.3(1) & 10.5(3) \end{pmatrix}$	$\begin{pmatrix} 6.1(2) & -1.5(2) \\ -0.4(1) & 9.6(2) \end{pmatrix}$	$\begin{pmatrix} 6.0(2) & -0.8(2) \\ -0.4(1) & 9.7(3) \end{pmatrix}$
4	0.500	0.250	$\begin{pmatrix} 3.8(1) & -2.3(3) \\ 1.5(1) & 9.4(3) \end{pmatrix}$	$\begin{pmatrix} 4.6(1) & -1.5(3) \\ -0.0(1) & 8.7(3) \end{pmatrix}$	$\begin{pmatrix} 4.6(1) & -0.8(3) \\ 0.2(1) & 8.7(3) \end{pmatrix}$
5	0.625	0.125	$\begin{pmatrix} 3.0(1) & -3.1(3) \\ 0.8(1) & 8.3(3) \end{pmatrix}$	$\begin{pmatrix} 3.5(1) & -2.8(3) \\ -0.0(1) & 7.8(3) \end{pmatrix}$	$\begin{pmatrix} 3.5(1) & -1.5(3) \\ 0.0(1) & 7.8(3) \end{pmatrix}$
6	0.250	0.250	$\begin{pmatrix} 5.8(2) & -1.0(2) \\ 0.4(1) & 9.4(3) \end{pmatrix}$	$\begin{pmatrix} 6.0(3) & -0.6(2) \\ -0.4(1) & 9.3(3) \end{pmatrix}$	$\begin{pmatrix} 6.0(2) & -0.3(2) \\ -0.6(1) & 9.3(3) \end{pmatrix}$

TABLE S3. Fick diffusion coefficient matrix in the molar, volume and mass reference frame of the ternary subsystem water (1) + ethanol (2) + 2-propanol (3) at 298.15 K and 0.1 MPa. The numbers in parentheses indicate the uncertainty in the last given digit.

No.	x_1	x_2	$\mathbf{D}^M$	$\mathbf{D}^V$	$\mathbf{D}^w$
	mol·mol^{-1}		10^{-10}m^{2}s^{-1}	10^{-10}m^{2}s^{-1}	10^{-10}m^{2}s^{-1}
1	0.125	0.625	$\begin{pmatrix} 6.6(2) & -0.4(1) \\ 0.2(2) & 8.0(2) \end{pmatrix}$	$\begin{pmatrix} 6.8(2) & -0.3(1) \\ -0.5(2) & 7.8(2) \end{pmatrix}$	$\begin{pmatrix} 6.8(2) & -0.1(1) \\ -1.0(2) & 7.8(2) \end{pmatrix}$
2	0.250	0.500	$\begin{pmatrix} 5.3(2) & -0.3(2) \\ 0.8(2) & 7.0(2) \end{pmatrix}$	$\begin{pmatrix} 5.5(2) & -0.2(2) \\ 0.0(2) & 6.9(2) \end{pmatrix}$	$\begin{pmatrix} 5.4(2) & -0.1(2) \\ 0.3(2) & 6.9(2) \end{pmatrix}$
3	0.375	0.375	$\begin{pmatrix} 4.4(1) & -0.5(2) \\ 0.6(1) & 6.2(2) \end{pmatrix}$	$\begin{pmatrix} 4.6(1) & -0.5(2) \\ 0.0(1) & 5.9(2) \end{pmatrix}$	$\begin{pmatrix} 4.6(1) & -0.2(2) \\ 0.2(1) & 6.0(2) \end{pmatrix}$
4	0.500	0.250	$\begin{pmatrix} 3.4(1) & -1.0(2) \\ 0.65(7) & 5.7(2) \end{pmatrix}$	$\begin{pmatrix} 3.7(1) & -1.1(2) \\ -0.00(5) & 5.4(2) \end{pmatrix}$	$\begin{pmatrix} 3.6(1) & -0.4(2) \\ 0.15(5) & 5.5(2) \end{pmatrix}$
5	0.625	0.125	$\begin{pmatrix} 2.84(8) & -1.1(2) \\ 0.61(4) & 5.8(2) \end{pmatrix}$	$\begin{pmatrix} 3.07(9) & -1.4(3) \\ 0.05(3) & 5.6(2) \end{pmatrix}$	$\begin{pmatrix} 3.05(9) & -0.5(2) \\ 0.25(3) & 5.6(2) \end{pmatrix}$
6	0.250	0.250	$\begin{pmatrix} 4.7(2) & -0.5(2) \\ 0.3(1) & 6.9(2) \end{pmatrix}$	$\begin{pmatrix} 4.8(2) & -0.4(2) \\ -0.2(1) & 6.8(2) \end{pmatrix}$	$\begin{pmatrix} 4.7(2) & -0.2(2) \\ -0.4(1) & 6.8(2) \end{pmatrix}$

TABLE S4. Fick diffusion coefficient matrix in the volume and mass reference frame of the quaternary mixture water (1) + methanol (2) + ethanol (3) + 2-propanol (4) at 298.15 K and 0.1 MPa. The numbers in parentheses indicate the uncertainty in the last given digit.

No.	x_1	x_2	x_3	$\mathbf{D}^V$	$\mathbf{D}^w$
	mol·mol^{-1}			10^{-10}m^{2}s^{-1}	10^{-10}m^{2}s^{-1}
1	0.125	0.5	0.125	$\begin{pmatrix} 10.1(2) & -1.5(2) & -0.5(2) \\ -0.4(2) & 12.7(2) & 0.0(3) \\ -0.7(2) & -1.4(2) & 10.9(2) \end{pmatrix}$	$\begin{pmatrix} 10.1(2) & -2.1(2) & -0.7(2) \\ -0.3(2) & 12.3(2) & -0.7(3) \\ -0.3(2) & -0.9(1) & 11.3(2) \end{pmatrix}$
2	0.125	0.375	0.25	$\begin{pmatrix} 9.0(2) & -1.2(2) & -0.5(2) \\ -0.7(3) & 12.5(2) & -0.1(2) \\ -0.5(2) & -0.5(2) & 10.8(2) \end{pmatrix}$	$\begin{pmatrix} 9.0(2) & -1.9(2) & -0.9(2) \\ -0.5(3) & 12.4(2) & -0.8(2) \\ -0.2(3) & -0.3(2) & 10.9(2) \end{pmatrix}$
3	0.125	0.25	0.375	$\begin{pmatrix} 8.2(2) & -0.1(2) & -0.6(2) \\ -0.7(2) & 10.8(3) & 0.1(3) \\ -0.4(2) & -0.5(3) & 8.9(2) \end{pmatrix}$	$\begin{pmatrix} 8.1(2) & -0.1(3) & -1.6(3) \\ -0.4(2) & 10.7(3) & -0.2(2) \\ -0.1(2) & -0.4(4) & 9.0(2) \end{pmatrix}$
4	0.125	0.125	0.5	$\begin{pmatrix} 7.4(2) & -0.5(2) & -0.6(2) \\ -0.6(2) & 10.8(2) & -0.4(2) \\ -0.6(2) & 0.1(3) & 8.7(2) \end{pmatrix}$	$\begin{pmatrix} 7.4(2) & -0.8(2) & -1.2(2) \\ 0.4(2) & 10.8(2) & -0.8(2) \\ -0.2(3) & 0.1(3) & 8.7(2) \end{pmatrix}$
5	0.25	0.375	0.125	$\begin{pmatrix} 7.5(1) & -1.8(3) & 0.2(2) \\ -0.6(1) & 11.3(2) & 0.2(3) \\ -0.6(2) & -1.0(1) & 8.7(2) \end{pmatrix}$	$\begin{pmatrix} 7.5(1) & -2.7(2) & 1.9(3) \\ -0.5(1) & 11.0(2) & -1.0(3) \\ -0.2(1) & -0.7(1) & 9.0(2) \end{pmatrix}$
6	0.25	0.25	0.25	$\begin{pmatrix} 6.7(2) & -0.2(2) & -0.2(2) \\ -0.9(2) & 10.1(3) & 0.0(2) \\ -0.5(2) & -0.4(4) & 8.3(2) \end{pmatrix}$	$\begin{pmatrix} 6.7(2) & -0.1(3) & -0.6(2) \\ -0.6(2) & 10.0(3) & -0.8(2) \\ -0.2(2) & -0.2(4) & 8.4(2) \end{pmatrix}$
7	0.25	0.125	0.375	$\begin{pmatrix} 6.2(1) & 0.7(3) & -0.5(2) \\ -0.3(1) & 10.1(2) & -0.6(1) \\ -0.5(1) & -0.4(3) & 7.7(1) \end{pmatrix}$	$\begin{pmatrix} 6.1(1) & 1.4(3) & -1.7(2) \\ -0.2(1) & 10.1(2) & -1.6(1) \\ -0.2(2) & -0.2(3) & 7.7(1) \end{pmatrix}$
8	0.375	0.25	0.125	$\begin{pmatrix} 5.5(1) & -0.9(3) & 0.3(3) \\ -0.8(1) & 10.0(2) & 0.5(2) \\ -1.2(1) & -0.3(2) & 7.8(2) \end{pmatrix}$	$\begin{pmatrix} 5.6(1) & -1.4(3) & 1.4(3) \\ -0.7(1) & 9.9(2) & -0.7(2) \\ -0.4(1) & -0.2(1) & 7.8(2) \end{pmatrix}$
9	0.375	0.125	0.25	$\begin{pmatrix} 4.9(1) & 0.6(3) & -0.3(2) \\ -0.5(1) & 8.8(2) & -0.1(1) \\ -1.2(1) & -0.4(2) & 7.1(2) \end{pmatrix}$	$\begin{pmatrix} 4.9(1) & -1.2(3) & -1.4(2) \\ -0.5(2) & 8.6(2) & -0.9(1) \\ -0.5(1) & -0.3(2) & 7.2(2) \end{pmatrix}$
10	0.5	0.125	0.125	$\begin{pmatrix} 4.1(1) & -0.1(3) & 0.1(3) \\ -1.2(1) & 8.6(2) & 0.8(2) \\ -0.9(2) & -0.6(1) & 6.1(1) \end{pmatrix}$	$\begin{pmatrix} 4.1(1) & -0.0(3) & 0.2(3) \\ -0.9(2) & 8.3(2) & -0.6(2) \\ -0.3(1) & -0.4(1) & 6.4(1) \end{pmatrix}$

TABLE S5. Fick diffusion coefficient matrix in the molar reference frame of the quaternary mixture water (1) + methanol (2) + ethanol (3) + 2-propanol (4) for varying component order at 298.15 K and 0.1 MPa. The numbers in parentheses indicate the uncertainty in the last given digit.

No.	$\mathbf{D}^M_{2341}$ $10^{-10}\mathrm{m^2s^{-1}}$	$\mathbf{D}^M_{1342}$ $10^{-10}\mathrm{m^2s^{-1}}$	$\mathbf{D}^M_{1243}$ $10^{-10}\mathrm{m^2s^{-1}}$
1	$\begin{pmatrix} 12.7(2) & -0.5(2) & 0.1(2) \\ 0.1(2) & 11.7(2) & 0.3(2) \\ -1.6(2) & -0.6(2) & 9.4(2) \end{pmatrix}$	$\begin{pmatrix} 10.7(2) & 0.2(2) & 0.9(2) \\ 0.3(3) & 12.0(2) & 0.6(2) \\ 1.6(2) & 1.0(2) & 11.0(2) \end{pmatrix}$	$\begin{pmatrix} 10.4(2) & -0.2(2) & 0.7(2) \\ 0.5(3) & 13.2(2) & 0.6(2) \\ 0.6(2) & -1.0(2) & 10.0(2) \end{pmatrix}$
2	$\begin{pmatrix} 12.6(2) & -0.2(2) & -0.1(2) \\ 0.5(2) & 11.1(2) & 0.0(3) \\ -2.1(2) & -1.5(2) & 8.7(2) \end{pmatrix}$	$\begin{pmatrix} 9.9(2) & 0.6(2) & 1.2(2) \\ 0.6(2) & 11.7(2) & 0.7(3) \\ 2.1(2) & 0.5(2) & 10.8(2) \end{pmatrix}$	$\begin{pmatrix} 9.2(2) & -0.6(2) & 0.7(2) \\ 0.2(2) & 12.8(2) & 0.1(2) \\ 1.5(2) & -0.5(3) & 10.3(2) \end{pmatrix}$
3	$\begin{pmatrix} 10.4(1) & -0.7(2) & -0.4(3) \\ 0.2(2) & 9.5(2) & 0.4(2) \\ -1.7(2) & -0.5(2) & 7.9(2) \end{pmatrix}$	$\begin{pmatrix} 8.9(1) & 0.6(2) & 1.0(2) \\ -0.1(2) & 9.4(2) & 0.3(2) \\ 1.7(2) & 1.1(2) & 9.6(2) \end{pmatrix}$	$\begin{pmatrix} 8.3(1) & -0.6(2) & 0.4(2) \\ 0.7(2) & 11.1(2) & 0.3(2) \\ 0.5(2) & -1.1(2) & 8.4(2) \end{pmatrix}$
4	$\begin{pmatrix} 10.9(2) & 0.2(3) & -0.1(3) \\ 0.9(2) & 8.7(2) & -0.2(2) \\ -1.9(2) & -1.2(3) & 7.3(2) \end{pmatrix}$	$\begin{pmatrix} 8.0(2) & 0.4(3) & 1.0(3) \\ 0.9(2) & 9.6(2) & 0.7(2) \\ 1.9(2) & 0.7(2) & 9.2(2) \end{pmatrix}$	$\begin{pmatrix} 7.7(1) & -0.4(3) & 0.7(2) \\ -0.2(2) & 10.7(2) & -0.2(2) \\ 1.2(2) & -0.7(2) & 8.5(2) \end{pmatrix}$
5	$\begin{pmatrix} 11.7(2) & 0.0(3) & 0.4(2) \\ 0.2(2) & 8.2(2) & -1.5(3) \\ -0.7(2) & -0.4(2) & 7.6(2) \end{pmatrix}$	$\begin{pmatrix} 8.5(1) & 0.7(3) & 1.3(2) \\ 1.7(2) & 9.9(2) & 0.8(3) \\ 1.5(2) & 1.1(2) & 9.1(2) \end{pmatrix}$	$\begin{pmatrix} 7.8(2) & -0.7(3) & 0.6(2) \\ 0.0(3) & 11.7(2) & 0.5(2) \\ 0.4(2) & -1.1(2) & 8.0(2) \end{pmatrix}$
6	$\begin{pmatrix} 9.7(2) & -1.5(1) & -0.7(2) \\ 0.6(2) & 8.5(2) & 0.0(3) \\ -1.3(2) & -0.8(3) & 7.0(2) \end{pmatrix}$	$\begin{pmatrix} 7.8(2) & 0.9(2) & 1.5(1) \\ 0.6(2) & 9.1(2) & 0.6(2) \\ 1.3(2) & 0.4(3) & 8.2(2) \end{pmatrix}$	$\begin{pmatrix} 6.9(2) & -0.9(2) & 0.6(2) \\ 0.8(1) & 10.4(2) & 0.1(2) \\ 0.8(2) & -0.4(3) & 7.8(2) \end{pmatrix}$
7	$\begin{pmatrix} 9.0(2) & -1.5(3) & -1.3(2) \\ 0.4(1) & 8.4(2) & 0.5(2) \\ -1.3(1) & -0.6(2) & 6.6(2) \end{pmatrix}$	$\begin{pmatrix} 6.7(1) & 0.5(2) & 0.9(2) \\ 1.0(1) & 9.3(2) & 1.5(1) \\ 1.3(1) & 0.7(2) & 7.9(2) \end{pmatrix}$	$\begin{pmatrix} 6.4(2) & -0.4(3) & 0.5(2) \\ 1.5(1) & 10.4(2) & 0.1(2) \\ 0.6(2) & -0.7(2) & 7.2(2) \end{pmatrix}$
8	$\begin{pmatrix} 10.1(2) & -1.5(2) & -0.5(3) \\ 1.3(1) & 7.0(2) & -1.0(2) \\ -1.6(1) & -0.8(2) & 6.1(2) \end{pmatrix}$	$\begin{pmatrix} 7.1(1) & 1.5(1) & 2.0(3) \\ 1.3(1) & 8.4(2) & 0.5(2) \\ 1.6(1) & 0.8(2) & 7.8(2) \end{pmatrix}$	$\begin{pmatrix} 6.2(2) & -0.9(2) & 1.1(2) \\ 0.0(3) & 10.1(2) & -0.1(2) \\ 0.8(2) & -0.8(2) & 7.0(2) \end{pmatrix}$
9	$\begin{pmatrix} 7.7(2) & -1.5(2) & -1.3(2) \\ 1.0(2) & 7.7(2) & 0.3(2) \\ -1.3(1) & -0.8(2) & 5.4(2) \end{pmatrix}$	$\begin{pmatrix} 5.9(1) & 0.6(3) & 1.6(2) \\ 0.4(2) & 8.1(2) & 0.7(2) \\ 1.3(1) & 0.5(2) & 6.7(2) \end{pmatrix}$	$\begin{pmatrix} 5.4(2) & -0.5(2) & 1.1(2) \\ 1.4(1) & 9.1(2) & 0.1(2) \\ 0.8(2) & -0.5(2) & 6.2(2) \end{pmatrix}$
10	$\begin{pmatrix} 8.1(2) & -1.5(1) & -0.5(2) \\ 0.5(2) & 6.1(2) & -0.4(3) \\ -1.4(1) & -0.7(2) & 4.7(2) \end{pmatrix}$	$\begin{pmatrix} 6.0(1) & -0.1(2) & 2.3(3) \\ -1.2(1) & 6.7(2) & 0.3(2) \\ 1.4(2) & 0.6(2) & 6.0(1) \end{pmatrix}$	$\begin{pmatrix} 4.5(2) & -1.5(1) & 0.8(2) \\ 0.9(2) & 8.9(2) & 0.3(2) \\ 0.7(2) & -0.6(2) & 5.4(2) \end{pmatrix}$

TABLE S6. Shear viscosity from a fit of simulated data for the quaternary mixture water (1) + methanol (2) + ethanol (3) + 2-propanol (4) at 298.15 K and 0.1 MPa in comparison with experimental literature data [1].

No.	x_1	x_2	x_3	η^{calc}	$\eta^{\exp}$
		mol·mol^{-1}		10^{-4}Pa · s	10^{-4}Pa · s
1	0.1988	0.1249	0.4999	15.75	15.24
2	0.2234	0.2638	0.2846	14.41	14.56
3	0.2913	0.4800	0.1054	12.89	13.26
4	0.3014	0.2433	0.3822	15.78	15.05
5	0.3182	0.3166	0.1911	15.16	15.76
6	0.3856	0.4473	0.0876	14.20	14.97
7	0.3918	0.2234	0.2440	17.31	17.46
8	0.4288	0.2830	0.2142	17.02	16.67
9	0.5158	0.1859	0.1846	19.67	19.76
10	0.5179	0.1725	0.1983	19.92	20.12
11	0.6021	0.1337	0.1264	21.91	23.37

II. FICK DIFFUSION COEFFICIENT MATRIX

Asymptotic behavior of the ternary Fick diffusion matrix

The asymptotic behavior towards the binary limits of the ternary Fick diffusion coefficient matrix can be obtained by comparing the diffusive flux equations of the ternary and binary mixtures. If one of the first two components is vanished, the following limits are given:
For $x_1 \to 0$

- $D_{12} \to 0$,
- $D_{22} \to D_{23}^{\text{bin}}$.

For $x_2 \to 0$

- $D_{21} \to 0$,
- $D_{11} \to D_{13}^{\text{bin}}$.

Therein, D_{ij} is the element in the ith row and the jth column of the ternary Fick diffusion coefficient matrix and D_{ij}^{bin} is the Fick diffusion coefficient of the binary subsystem $i + j$.

Frame of reference

The Fick diffusion coefficient matrix depends on the velocity reference frame. The molar reference frame is the natural choice for molecular simulations, but experimental data are typically evaluated on the volume reference frame. On the other hand, in non-equilibrium thermodynamics, usually the mass reference frame is assumed. To compare among different approaches, it is important to be aware how the according Fick diffusion coefficients relate to each other. The Fick diffusion coefficient matrix in the molar reference frame $\mathbf{D}^M$ can be transformed into its form in the mass reference frame $\mathbf{D}^w$ employing [2]

$$[\mathbf{D}^w] = [\mathbf{B}^{wu}][\mathbf{w}][\mathbf{x}]^{-1}[\mathbf{D}^M][\mathbf{x}][\mathbf{w}]^{-1}[\mathbf{B}^{uw}], \tag{S1}$$

where $[\mathbf{x}]$ and $[\mathbf{w}]$ are the diagonal matrices of the mole and mass fractions x_i and w_i, respectively. The elements of the matrices $\mathbf{B}^{wu}$ and $\mathbf{B}^{uw}$ are given by

$$\begin{aligned}B^{wu}_{ik} &= \delta_{ik} - w_i\left(1 - \frac{w_n x_k}{x_n w_k}\right),\\ B^{uw}_{ik} &= \delta_{ik} - w_i\left(\frac{x_k}{w_k} - \frac{x_n}{w_n}\right).\end{aligned} \tag{S2}$$

where δ_{ik} is the Kronecker delta and the lower index n refers to the solvent. Similarly, $\mathbf{D}^M$ can be transformed to the volume reference frame $\mathbf{D}^V$ with

$$[\mathbf{D}^V] = [\mathbf{B}^{Vu}][\mathbf{D}^M][\mathbf{B}^{uV}], \tag{S3}$$

and

$$\begin{aligned}B^{Vu}_{ik} &= \delta_{ik} - x_i\left(v_k - v_n\right)/v,\\ B^{uV}_{ik} &= \delta_{ik} - x_i\left(1 - v_k/v_n\right),\end{aligned} \tag{S4}$$

where the total molar volume v is the weighted sum of the partial molar volumes v_i

$$v = \sum_{i=1}^{n} x_i v_i. \tag{S5}$$

Choice of the solvent

In a multicomponent mixture, there are several ways to order the components. Usually, the species with the highest concentration is chosen as the solvent (s) due to accuracy concerns. However, the choice of component numbering is arbitrary.

The Fick approach for quaternary mixtures involves three independent molar diffusion fluxes

$$J_1/c_t = -D_{11}\nabla x_1 - D_{12}\nabla x_2 - D_{13}\nabla x_3, \tag{S6}$$
$$J_2/c_t = -D_{21}\nabla x_1 - D_{22}\nabla x_2 - D_{31}\nabla x_3, \tag{S7}$$
$$J_3/c_t = -D_{31}\nabla x_1 - D_{32}\nabla x_2 - D_{33}\nabla x_3, \tag{S8}$$

where the solvent component is s=4, c_t is the mixture molar density and D_{ij} are the elements of the Fick diffusion matrix in the molar reference frame. Varying component order changes the values of the Fick diffusion coefficient matrix, but not the actual molar fluxes. Since $\sum_{i=1}^{4} J_i = 0$ and $\nabla x_1 + \nabla x_2 + \nabla x_3 + \nabla x_4 = 0$, it follows for:

$s = 1$

$$J_2/c_t = -(D_{22} - D_{21})\nabla x_2 - (D_{23} - D_{21})\nabla x_3 - (-D_{21})\nabla x_4, \tag{S9}$$
$$J_3/c_t = -(D_{32} - D_{31})\nabla x_2 - (D_{33} - D_{31})\nabla x_3 - (-D_{31})\nabla x_4, \tag{S10}$$
$$\begin{aligned}J_4/c_t = &-(D_{11} + D_{21} + D_{31} - D_{12} - D_{22} - D_{32})\nabla x_2,\\ &-(D_{11} + D_{21} + D_{31} - D_{13} - D_{23} - D_{33})\nabla x_3,\\ &-(D_{11} + D_{21} + D_{31})\nabla x_4.\end{aligned} \tag{S11}$$

$s = 2$

$$J_1/c_t = -(D_{11} - D_{12})\nabla x_1 - (D_{13} - D_{12})\nabla x_3 - (-D_{12})\nabla x_4, \tag{S12}$$

$$J_3/c_t = -(D_{31} - D_{32})\nabla x_1 - (D_{33} - D_{32})\nabla x_3 - (-D_{32})\nabla x_4, \tag{S13}$$

$$\begin{aligned} J_4/c_t = &-(D_{12} + D_{22} + D_{32} - D_{11} - D_{21} - D_{31})\nabla x_1, \\ &-(D_{12} + D_{22} + D_{32} - D_{13} - D_{23} - D_{33})\nabla x_3, \\ &-(D_{12} + D_{22} + D_{32})\nabla x_4. \end{aligned} \tag{S14}$$

$s = 3$

$$J_1/c_t = -(D_{11} - D_{13})\nabla x_1 - (D_{12} - D_{13})\nabla x_2 - (-D_{13})\nabla x_4, \tag{S15}$$

$$J_2/c_t = -(D_{21} - D_{23})\nabla x_1 - (D_{22} - D_{23})\nabla x_2 - (-D_{23})\nabla x_4, \tag{S16}$$

$$\begin{aligned} J_4/c_t = &-(D_{13} + D_{23} + D_{33} - D_{11} - D_{21} - D_{31})\nabla x_1, \\ &-(D_{13} + D_{23} + D_{33} - D_{12} - D_{22} - D_{32})\nabla x_2, \\ &-(D_{13} + D_{23} + D_{33})\nabla x_4. \end{aligned} \tag{S17}$$

III. MOLECULAR SIMULATION

Force fields

Molecular dynamics simulations rely on force fields that mimic the intermolecular interactions adequately. In this work, rigid and non-polarizable force fields of united-atom type were employed, which account for these interactions by a set of Lennard-Jones sites and point charges which may or may not coincide with respect to their site positions. The force fields for the three alcohols were developed by our group based on quantum chemical calculations, parameter optimization to experimental vapor-liquid equilibrium data and, in the case of 2-propanol, also to experimental self-diffusion coefficient data [3–6]. For water, the TIP4P/2005 force field by Abascal and Vega [3] was employed. This force field was found to predict the transport properties of water and aqueous alcoholic mixtures with a better accuracy than other commonly used non-polarizable force fields [7]. The employed force fields have been tested with respect to their ability to predict structural, thermodynamic and transport properties of the corresponding pure substances [6–8], some binary [6, 8, 9], ternary [9, 10] as well as their quaternary mixture [11]. Detailed information about these force fields can be found in Table S7 and the original publications [3–6].

To define a molecular model for a mixture on the basis of pairwise additive pure substance force fields, only the unlike interactions have to be specified. In case of the point charges, this can straightforwardly be done with Coulomb's law. However, for the unlike Lennard-Jones parameters, there is no physically sound approach so that combining rules have to be employed. The Lorentz-Berthelot combining rules were chosen here

$$\sigma_{ab} = (\sigma_{aa} + \sigma_{bb})/2,$$

and

$$\varepsilon_{ab} = \sqrt{\epsilon_{aa}\varepsilon_{bb}}\,,$$

so that all present mixture data are strictly predictive.

TABLE S7. Lennard-Jones and point charge parameters of the employed force fields, k_B and e are Boltzmann's constant and the electronic charge, respectively.

site	$\varepsilon/\ k_B\cdot\ K^{-1}$	σ/Å	q/e	site	$\varepsilon/\ k_B\cdot\ K^{-1}$	σ/Å	q/e
methanol				ethanol			
S_{CH3}	3.7543	120.592	+0.24746	S_{CH3}	3.6072	120.15	–
S_{OH}	3.0300	87.879	−0.67874	S_{CH2}	3.4612	86.291	+0.25560
S_H	–	–	+0.43128	S_{OH}	3.1496	85.053	−0.69711
				S_H	–	–	+0.44151
2-propanol				water			
S_{CH3}	103.59	3.866	0	S_O	93.2	3.1589	−1.1128
S_{CH3}	103.59	3.866	0	S_H	–	–	+0.5564
S_{CH}	20.20	3.238	0.310	S_H	–	–	+0.5564
S_{OH}	85.90	3.154	-0.747				
S_H	–	–	0.437				

Phenomenological coefficients

Transport data were sampled by equilibrium molecular dynamics simulation and the Green-Kubo formalism based on the net velocity auto-correlation function to obtain the phenomenological coefficient matrix [12]

$$L_{ij} = \frac{1}{3N} \int_0^\infty \mathrm{dt} \left\langle \sum_{k=1}^{N_i} \mathbf{v}_{i,k}(0) \cdot \sum_{l=1}^{N_j} \mathbf{v}_{j,l}(\mathrm{t}) \right\rangle . \tag{S18}$$

Here, N is the total number of molecules. N_i and N_j are the number of molecules of components i and j, respectively. $\mathbf{v}_{i,k}(t)$ is the center of mass velocity vector of the k-th molecule of component i at time t. The brackets <...> denote the canonical (NVT) ensemble average and equation (S18) corresponds to a reference frame in which the mass-averaged velocity of the mixture is zero [12].

With the phenomenological coefficients L_{ij}, the elements of a matrix $\mathbf{\Delta}$ can be defined [12]

$$\Delta_{ij} = (1 - x_i) \left(\frac{L_{ij}}{x_j} - \frac{L_{in}}{x_n} \right) - x_i \sum_{k=1 \neq i}^{n} \left(\frac{L_{kj}}{x_j} - \frac{L_{kn}}{x_n} \right) , \tag{S19}$$

which is related to the matrix $\mathbf{B}$ by its inverse, $\mathbf{B} = \mathbf{\Delta}^{-1}$.

In the case of a binary mixture, the Maxwell-Stefan diffusion coefficient $Đ$ can be calculated with

$$Đ = \frac{x_2}{x_1} L_{11} + \frac{x_1}{x_2} L_{22} - L_{12} - L_{21} . \tag{S20}$$

In the ternary case, the three Maxwell-Stefan diffusion coefficients are given by

$$\begin{aligned} Đ_{12} &= \frac{1}{B_{11} - B_{12} \cdot (x_1 + x_3)/x_1} , \\ Đ_{13} &= \frac{1}{B_{11} + B_{12} \cdot (x_2/x_1)} , \\ Đ_{23} &= \frac{1}{B_{22} + B_{21} \cdot (x_1/x_2)} . \end{aligned} \tag{S21}$$

Shear viscosity

The shear viscosity η was calculated concurrently with the phenomenological coefficients employing the Green-Kubo formalism. It is associated with the off-diagonal elements of the microscopic stress tensor J_p^{xy}

$$\eta = \frac{1}{V k_B T} \int_0^\infty dt \, \langle J_p^{xy}(t) \cdot J_p^{xy}(0) \rangle, \tag{S22}$$

where V is the volume, k_B is the Boltzmann constant and T die temperature.

$$J_p^{xy} = \sum_{k=1}^{N} m_k v_k^x v_k^y - \frac{1}{2} \sum_{k=1}^{N} \sum_{l \neq k}^{N} r_{kl}^x \frac{\partial u(r_{kl})}{\partial r_{kl}^y}. \tag{S23}$$

Here, k and l denote different molecules of any species. The upper indices x and y stand for the spatial vector components, e.g. for velocity v_k^x or site-site distance r_{kl}^x. $u(r_{kl})$ is intramolecular the potential energy. Equations (S22) and (S23) may directly be applied to mixtures. To improve statistics, five independent terms of the stress tensor J_p^{xy}, J_p^{xz}, J_p^{yz}, $(J_p^{xx} - J_p^{yy})/2$ and $(J_p^{yy} - J_p^{zz})/2$ were considered [13].

Technical details

Molecular dynamics simulations were performed with the program $ms2$ [14, 15] in two steps: First, a simulation in the isobaric-isothermal (NpT) ensemble was carried out to calculate the density at the desired temperature, pressure and composition. In the second step, a canonic (NVT) ensemble simulation was performed under the corresponding thermodynamic conditions to simultaneously determine the phenomenological coefficient and thermodynamic factor matrices. Newton's equations of motion were solved with a fifth-order Gear predictor-corrector numerical integrator and the temperature was controlled by velocity scaling. Throughout, the integration time step was 0.925 fs. The simulations contained 6000 molecules and were carried out in a cubic volume with periodic boundary conditions, where the cut-off radius was set to $r_c = 24.5$ Å. Lennard-Jones long range interactions were considered using angle averaging [16]. Electrostatic long-range corrections were approximated by the reaction field technique with conducting boundary conditions ($\epsilon_{RF} = \infty$).

The simulations in the NpT ensemble were equilibrated over 4×10^5 time steps, followed by a production run over 3×10^6 time steps. In the NVT ensemble, the simulations were equilibrated over 5×10^5 time steps, followed by production runs of 8×10^7 time steps. The phenomenological coefficients were calculated for up to 8×10^5 independent time origins of the correlation functions. The sampling length of the correlation functions was of 20 000 time steps or 18.5 ps throughout. The separation between the time origins was chosen such that all autocorrelation functions have decayed at least to $1/e$ of their normalized value to achieve their time independence [17]. The uncertainties of the predicted values were estimated with a block averaging method [18].

IV. FINITE SIZE EFFECTS

To asses finite size correction methods, simulations were performed for the three ternary subsystems methanol + ethanol + 2-propanol, water + methanol + 2-propanol and water + ethanol + 2-propanol with system sizes containing between 512 and 8000 molecules with composition $x_1 = 0.125$, $x_2 = 0.625$ and $x_3 = 0.25$ mol·mol^{-1} for all mixtures. Additionally, the ternary mixture water + ethanol + 2-propanol was regarded for the composition $x_1 = 0.25$, $x_2 = 0.5$ and $x_3 = 0.25$ mol·mol^{-1}. System size effects of the intra-, phenomenological, Maxwell-Stefan and Fick diffusion coefficients were evaluated by plotting the simulation results over the inverse of the edge length of the simulation volume L^{-1} and fitting a straight line to the data. The resulting intercept for $L^{-1} \to 0$ corresponds to the infinite size value.

Intra-diffusion coefficients

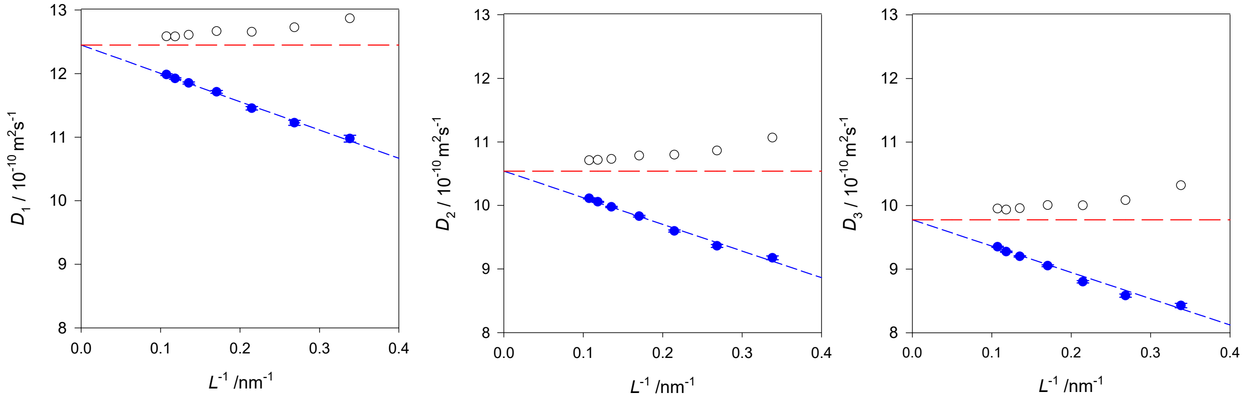

FIG. S2. Intra-diffusion coefficients of methanol (left), ethanol (center) and 2-propanol (right) in their ternary mixture ($x_{\mathrm{CH4O}} = 0.125$, $x_{\mathrm{C2H6O}} = 0.625$ and $x_{\mathrm{C3H8O}} = 0.25$ mol·mol^{-1}) as a function of the inverse edge length of the simulation volume L^{-1} at 298.15 K and 0.1 MPa. The uncorrected simulation results (blue bullets) are shown together with the values corrected with the approach by Yeh and Hummer (black circles). The blue dashed line is a linear fit to the uncorrected simulation results and the red line represents the extrapolated value in the thermodynamic limit.The error bars are within symbol size.

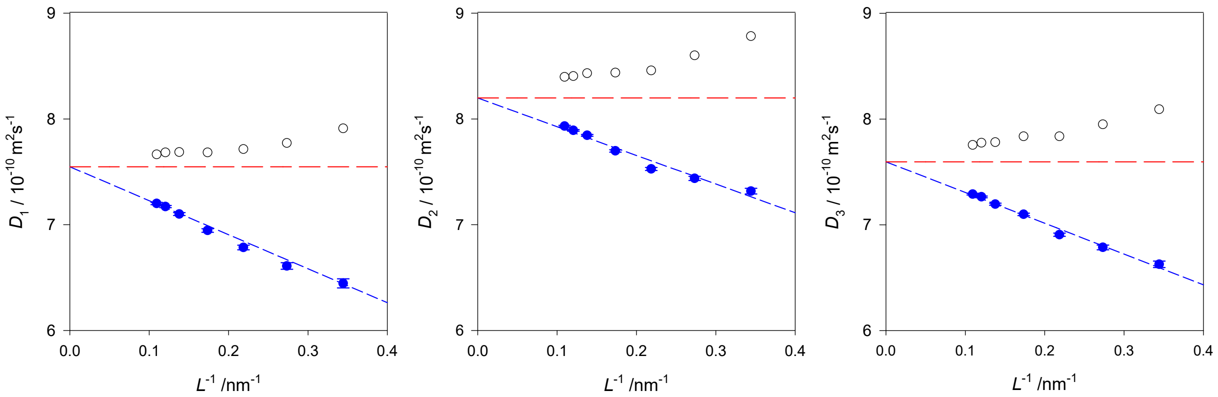

FIG. S3. Intra-diffusion coefficients of water (left), ethanol (center) and 2-propanol (right) in their ternary mixture ($x_{\mathrm{H2O}} = 0.125$, $x_{\mathrm{C2H6O}} = 0.625$ and $x_{\mathrm{C3H8O}} = 0.25$ mol·mol^{-1}) as a function of the inverse edge length of the simulation volume L^{-1} at 298.15 K and 0.1 MPa. The uncorrected simulation results (blue bullets) are shown together with the values corrected with the approach by Yeh and Hummer (black circles). The blue dashed line is a linear fit to the uncorrected simulation results and the red line represents the extrapolated value in the thermodynamic limit. The error bars are within symbol size.

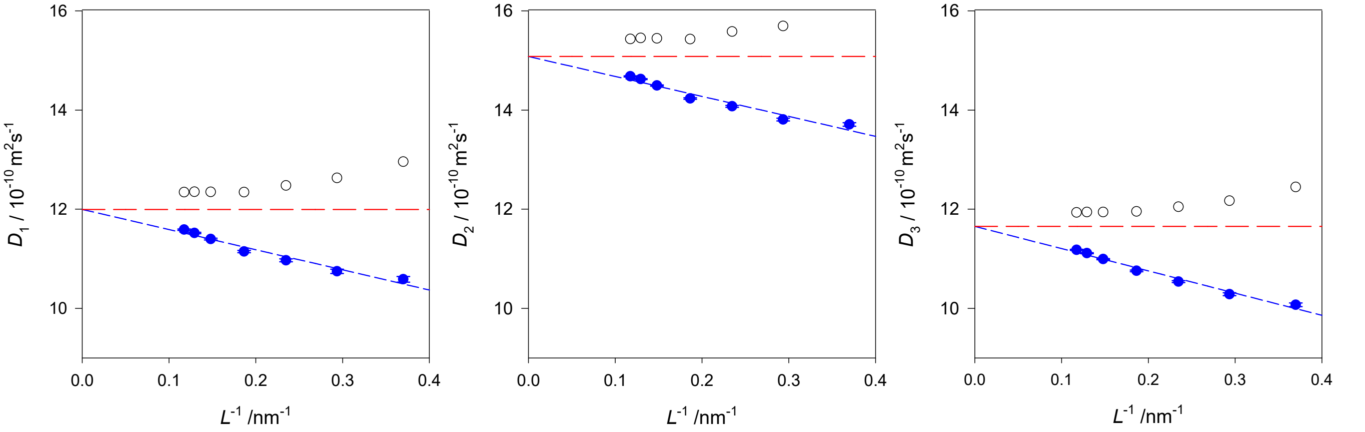

FIG. S4. Intra-diffusion coefficients of water (left), methanol (center) and 2-propanol (right) in their ternary mixture ($x_{\mathrm{H2O}} = 0.125$, $x_{\mathrm{CH4O}} = 0.625$ and $x_{\mathrm{C3H8O}} = 0.25$ mol·mol^{-1}) as a function of the inverse edge length of the simulation volume L at 298.15 K and 0.1 MPa. The uncorrected simulation results (blue bullets) are shown together with the corrected values using the YH approach (black circles). The blue dashed line is a linear fit to the uncorrected simulation results and the red line represents the extrapolated value in the thermodynamic limit. The error bars are within symbol size.

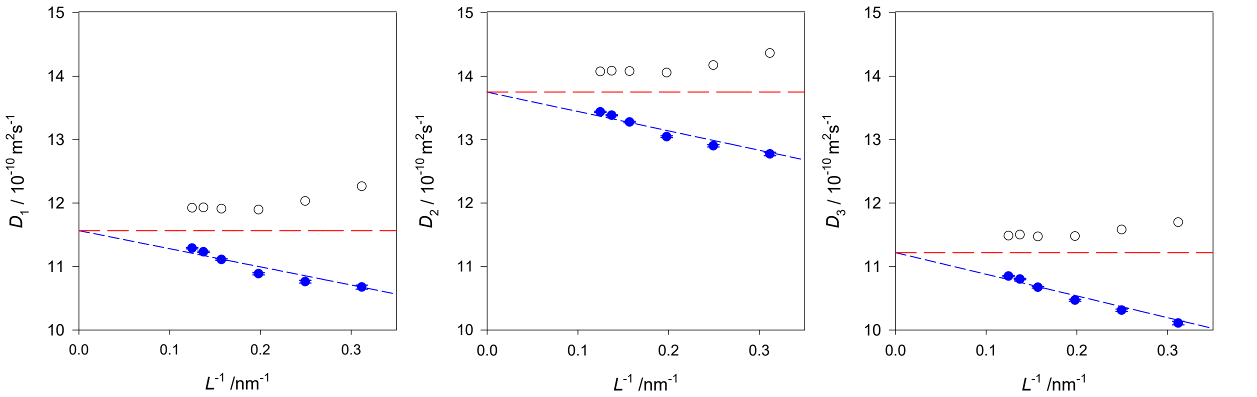

FIG. S5. Intra-diffusion coefficients of water (left), methanol (center) and 2-propanol (right) in their ternary mixture ($x_{\mathrm{H2O}} = 0.25$, $x_{\mathrm{CH4O}} = 0.5$ and $x_{\mathrm{C3H8O}} = 0.25$ mol·mol^{-1}) as a function of the inverse edge length of the simulation volume L^{-1} at 298.15 K and 0.1 MPa. The uncorrected simulation results (blue bullets) are shown together with the values corrected with the approach of Yeh and Hummer (black circles). The blue dashed line is a linear fit to the uncorrected simulation results and the red line represents the extrapolated value in the thermodynamic limit. The error bars are within symbol size.

Phenomenological coefficients

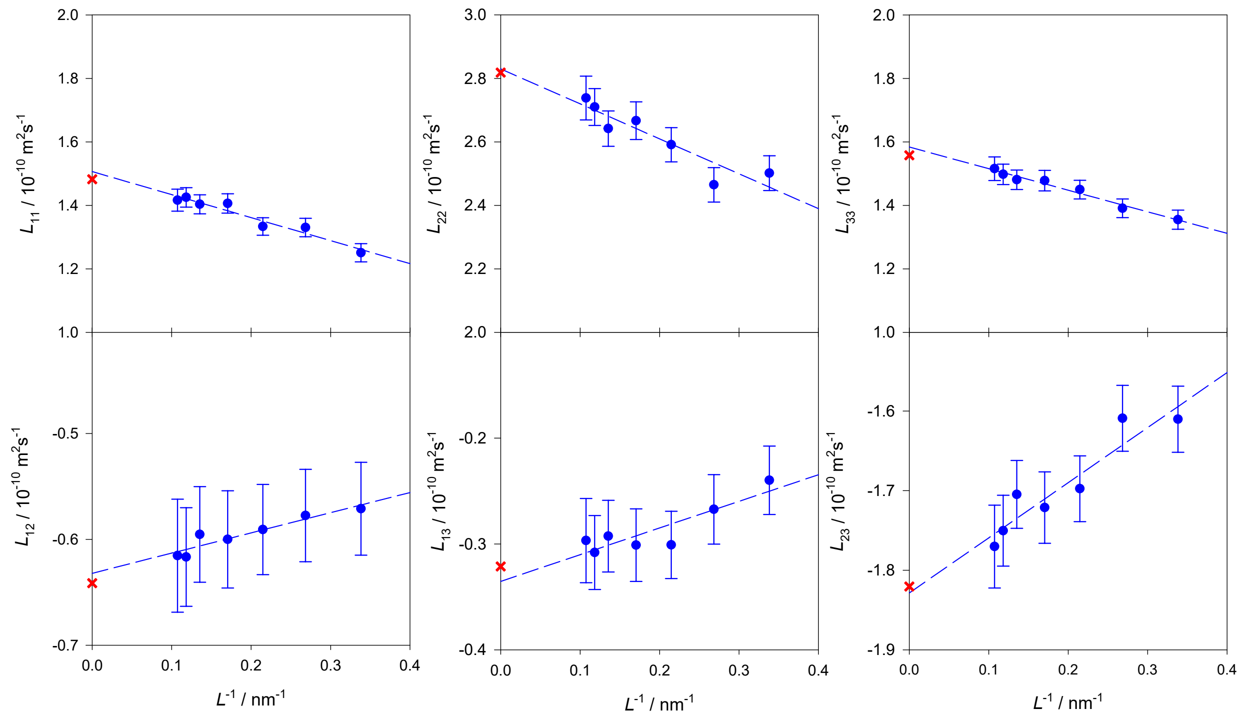

FIG. S6. Phenomenological coefficients of the ternary subsystem methanol (1) + ethanol (2) + 2-propanol (3) ($x_{\mathrm{CH4O}} = 0.125$, $x_{\mathrm{C2H6O}} = 0.625$ and $x_{\mathrm{C3H8O}} = 0.25$ mol·mol^{-1}) as a function of the inverse edge length of the simulation volume L^{-1} at 298.15 K and 0.1 MPa. The uncorrected simulation results (blue bullets) are shown together with the corrected values using the fast correction procedure [11] (red crosses). The blue dashed line is a linear fit to the uncorrected simulation results.

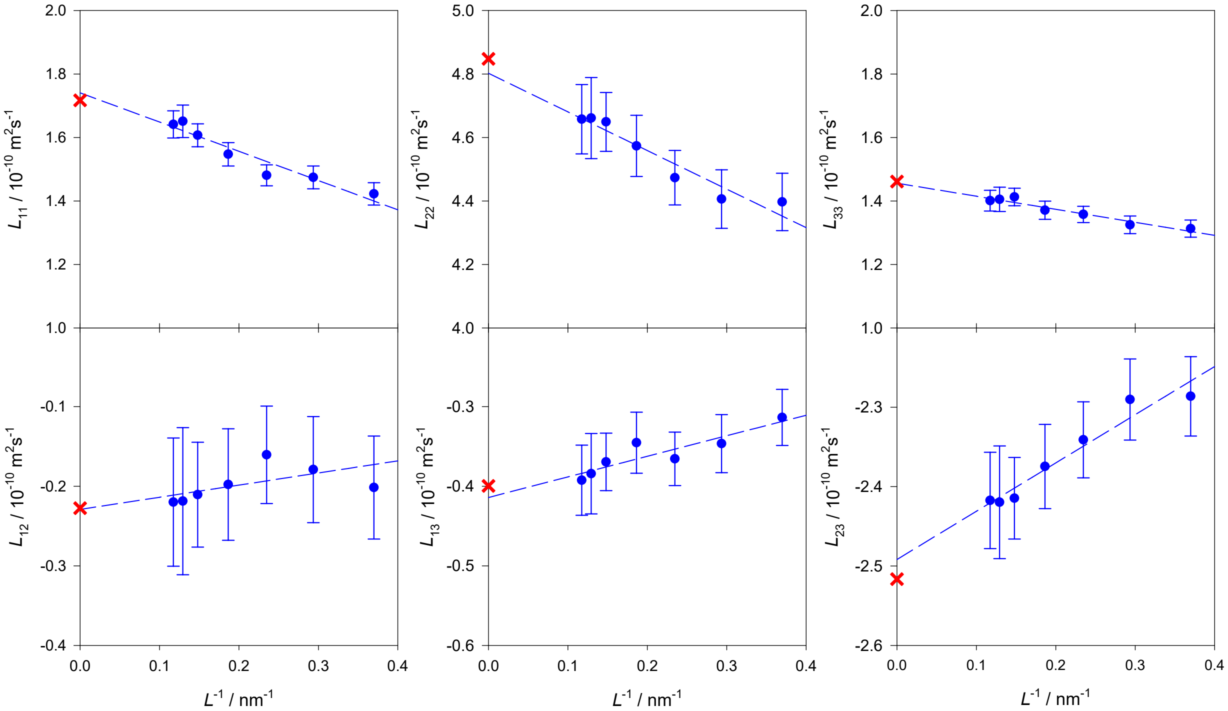

FIG. S7. Phenomenological coefficients of the ternary subsystem water (1) + methanol (2) + 2-propanol (3) ($x_{\mathrm{H2O}} = 0.125$, $x_{\mathrm{CH4O}} = 0.625$ and $x_{\mathrm{C3H8O}} = 0.25$ mol·mol^{-1}) as a function of the inverse edge length of the simulation volume L^{-1} at 298.15 K and 0.1 MPa. The uncorrected simulation results (blue bullets) are shown together with the corrected values using the fast correction procedure [11] (red crosses). The blue dashed line is a linear fit to the uncorrected simulation results.

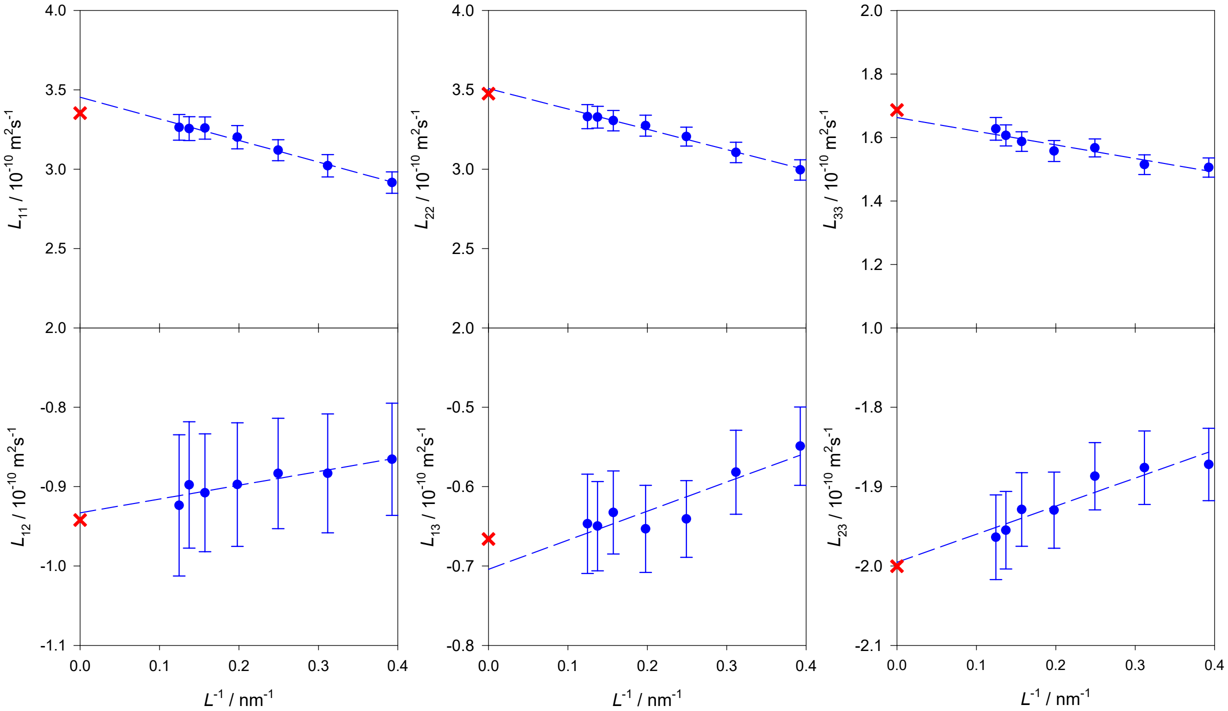

FIG. S8. Phenomenological coefficients of the ternary subsystem water (1) + methanol (2) + 2-propanol (3) ($x_{\mathrm{H2O}} = 0.25$, $x_{\mathrm{CH4O}} = 0.5$ and $x_{\mathrm{C3H8O}} = 0.25$ mol·mol^{-1}) as a function of the inverse edge length of the simulation volume L^{-1} at 298.15 K and 0.1 MPa. The uncorrected simulation results (blue bullets) are shown together with the corrected values using the fast correction procedure [11] (red crosses). The blue dashed line is a linear fit to the uncorrected simulation results.

Maxwell-Stefan diffusion coefficients

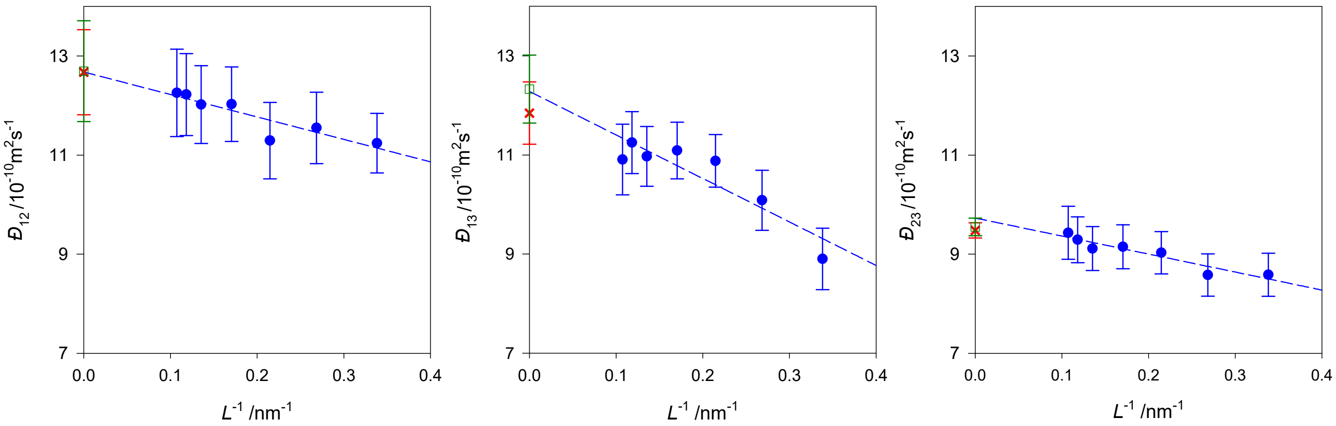

FIG. S9. Elements of the Maxwell-Stefan diffusion coefficient matrix of the ternary subsystem methanol (1) + ethanol (2) + 2-propanol (3) ($x_{\mathrm{CH4O}} = 0.125$, $x_{\mathrm{C2H6O}} = 0.625$ and $x_{\mathrm{C3H8O}} = 0.25$ mol·mol^{-1}) as a function of the inverse edge length of the simulation volume L^{-1} at 298.15 K and 0.1 MPa. The blue dashed line is a linear fit to the uncorrected simulation results (blue bullets). The coefficients calculated with the corrected values using the fast correction procedure [11] for $N = 6000$ (red crosses) and employing the individually extrapolated phenomenological coefficients (green squares) are also shown.

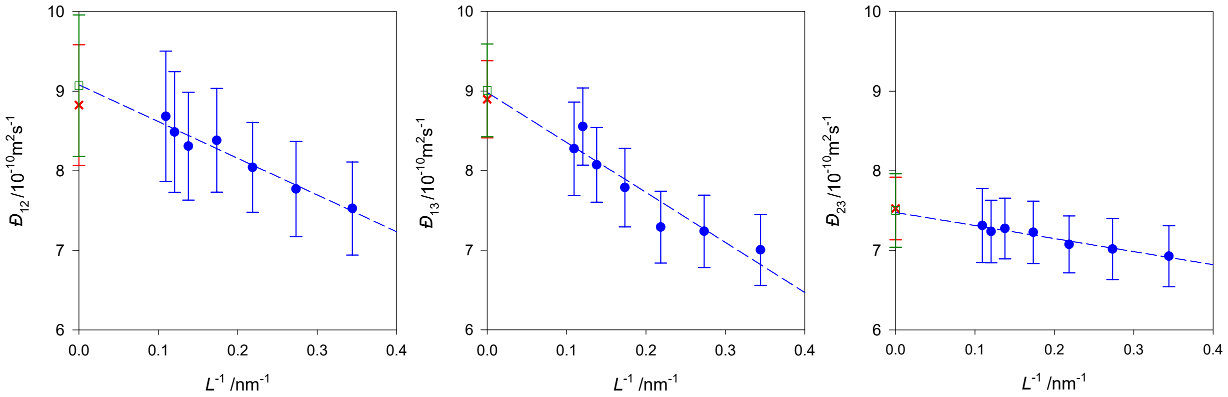

FIG. S10. Independent elements of Maxwell-Stefan diffusion coefficient matrix of the ternary subsystem water (1) + ethanol (2) + 2-propanol (3) ($x_{\mathrm{H2O}} = 0.125$, $x_{\mathrm{C2H6O}} = 0.625$ and $x_{\mathrm{C3H8O}} = 0.25$ mol·mol^{-1}) as a function of the inverse edge length of the simulation volume L^{-1} at 298.15 K and 0.1 MPa. The blue dashed line is a linear fit to the uncorrected simulation results (blue bullets). Coefficients subject to the fast correction procedure [11] (red crosses) and based on the individually extrapolated phenomenological coefficients (green squares) are shown.

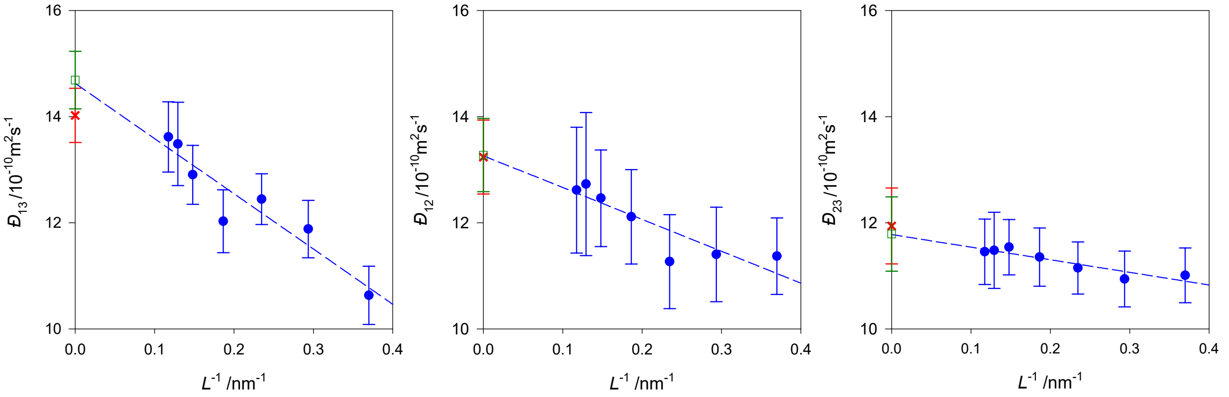

FIG. S11. Independent elements of Maxwell-Stefan diffusion coefficient matrix of the ternary subsystem water (1) + methanol (2) + 2-propanol (3) ($x_{\mathrm{H2O}} = 0.125$, $x_{\mathrm{CH4O}} = 0.625$ and $x_{\mathrm{C3H8O}} = 0.25$ mol·mol^{-1}) as a function of the inverse edge length of the simulation volume L^{-1} at 298.15 K and 0.1 MPa. The blue dashed line is a linear fit to the uncorrected simulation results (blue bullets). Coefficients subject to the fast correction procedure [11] (red crosses) and based on the individually extrapolated phenomenological coefficients (green squares) are shown.

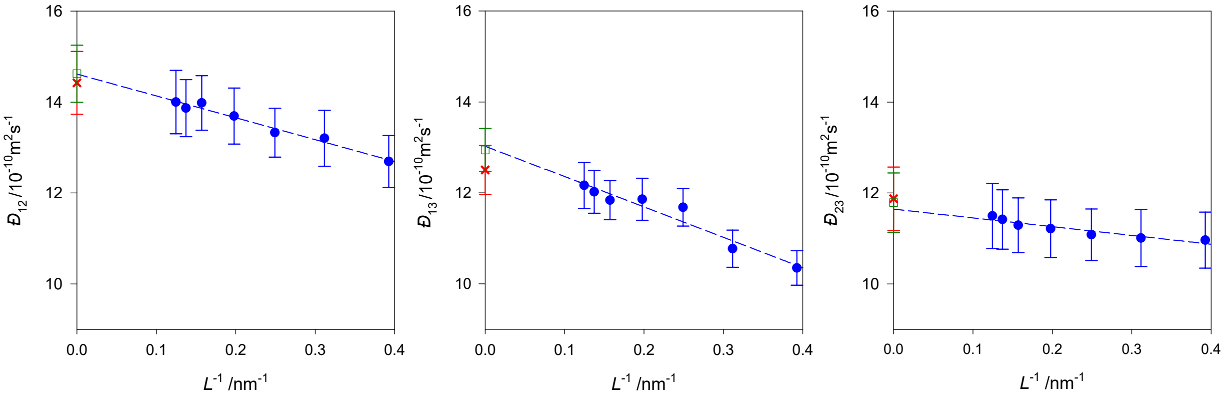

FIG. S12. Elements of Maxwell-Stefan diffusion coefficient matrix of the ternary subsystem water (1) + methanol (2) + 2-propanol (3) ($x_{\mathrm{H2O}} = 0.25$, $x_{\mathrm{CH4O}} = 0.5$ and $x_{\mathrm{C3H8O}} = 0.25$ mol·mol^{-1}) as a function of the inverse edge length of the simulation volume L^{-1} at 298.15 K and 0.1 MPa. The blue dashed line is a linear fit to the uncorrected simulation results (blue bullets). Coefficients subject to the fast correction procedure [11] (red crosses) and based on the individually extrapolated phenomenological coefficients (green squares) are shown.

Fick diffusion coefficient

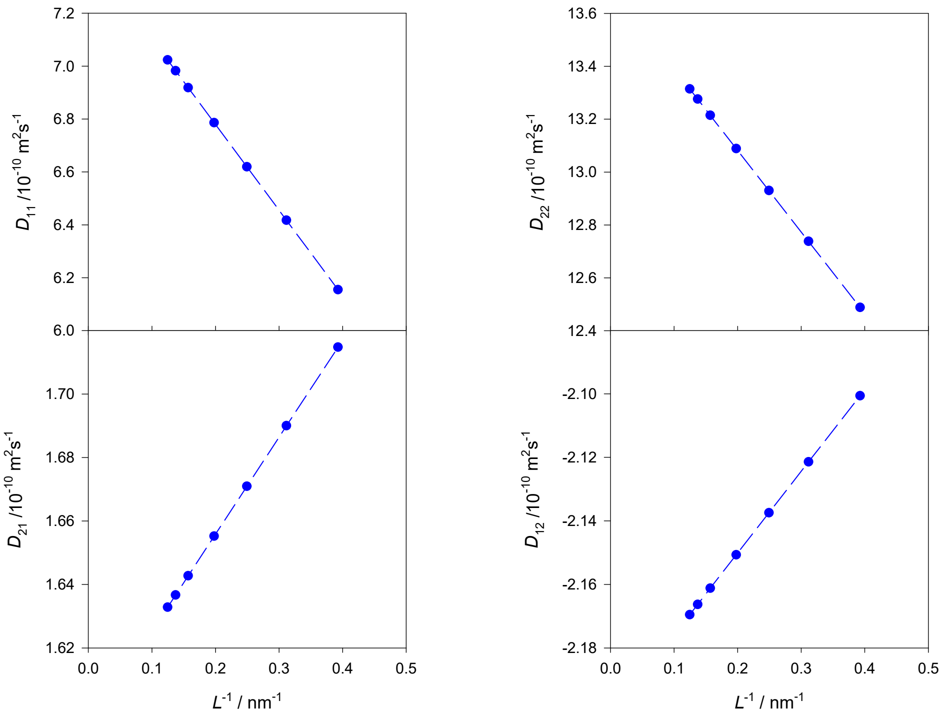

FIG. S13. Elements of the Fick diffusion coefficient matrix of the ternary subsystem water (1) + methanol (2) + 2-propanol (3) ($x_{\rm H2O} = 0.25$, $x_{\rm CH4O} = 0.5$ and $x_{\rm C3H8O} = 0.25$ mol·mol^{-1}) as a function of the inverse edge length of the simulation volume L^{-1} at 298.15 K and 0.1 MPa. The Fick diffusion coefficients were calculated on the basis of fitted values of the phenomenological coefficients L_{ij}.

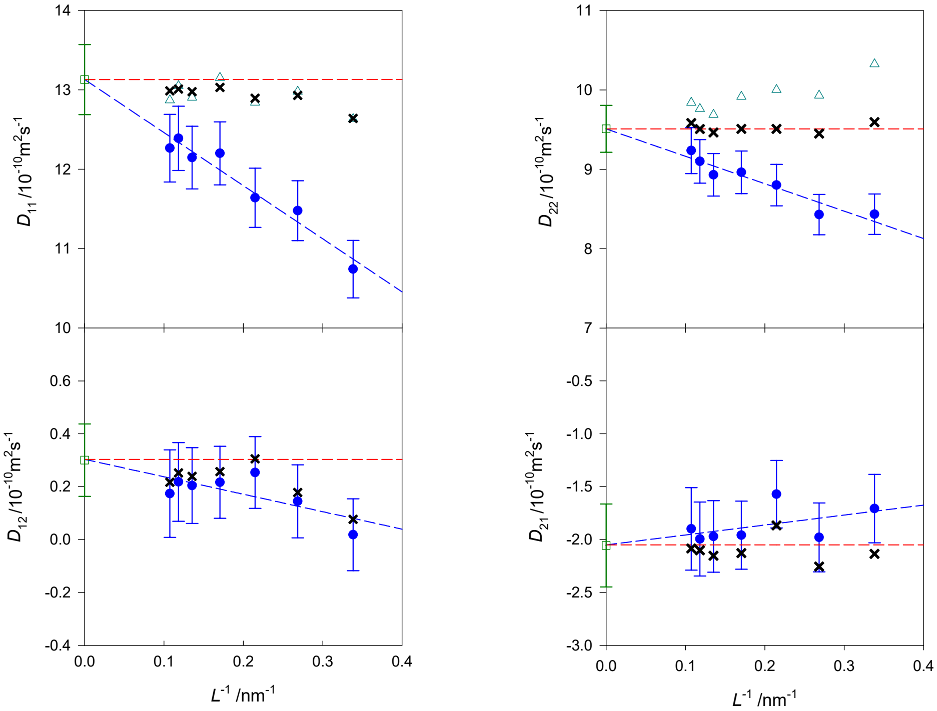

FIG. S14. Elements of the Fick diffusion coefficient matrix of the ternary subsystem methanol (1) + ethanol (2) + 2-propanol (3) ($x_{\mathrm{CH4O}} = 0.125$, $x_{\mathrm{C2H6O}} = 0.625$ and $x_{\mathrm{C3H8O}} = 0.25$ mol·mol^{-1}) as a function of the inverse edge length of the simulation volume L^{-1} at 298.15 K and 0.1 MPa. The blue dashed line is a linear fit to the uncorrected simulation results (blue bullets). Coefficients subject to the fast correction procedure [11] (crosses) are compared with those corrected with the procedure by Jamali et al. [19] (cyan triangles). The green squares represent the Fick diffusion coefficients based on the individually extrapolated phenomenological coefficients.

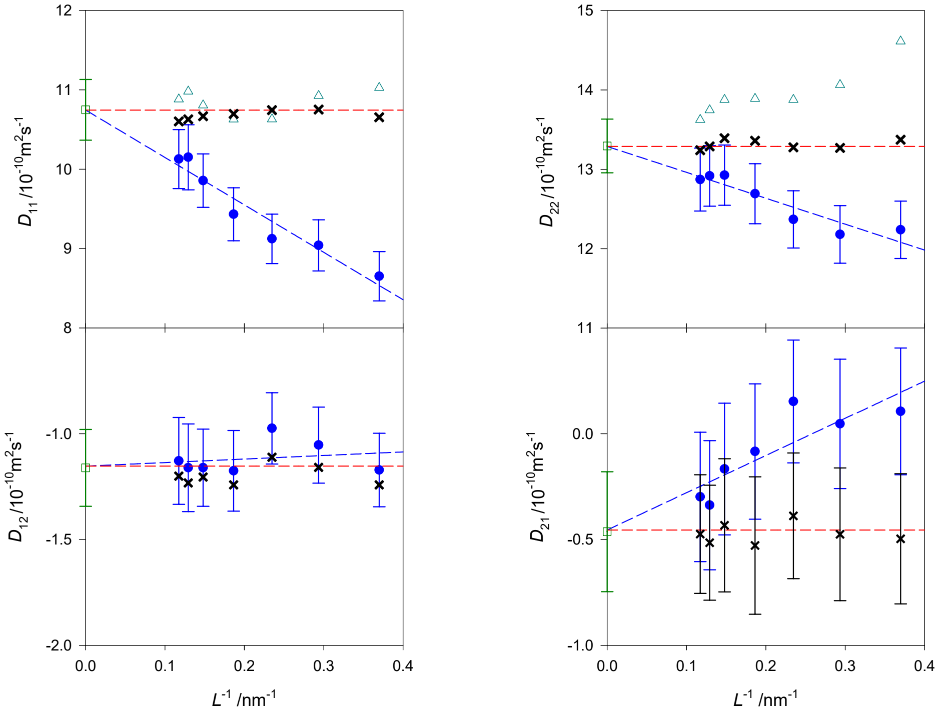

FIG. S15. Elements of the Fick diffusion coefficient matrix of the ternary subsystem water (1) + methanol (2) + 2-propanol (3) (x_{H2O} = 0.125, x_{CH4O} = 0.625 and x_{C3H8O} = 0.25 mol·mol^{-1}) as a function of the inverse edge length of the simulation volume L^{-1} at 298.15 K and 0.1 MPa. The blue dashed line is a linear fit to the uncorrected simulation results (blue bullets). Coefficients subject to the fast correction procedure [11] (crosses) are compared with those corrected with the procedure by Jamali et al. [19] (cyan triangles). The green squares represent the Fick diffusion coefficients based on the individually extrapolated phenomenological coefficients.

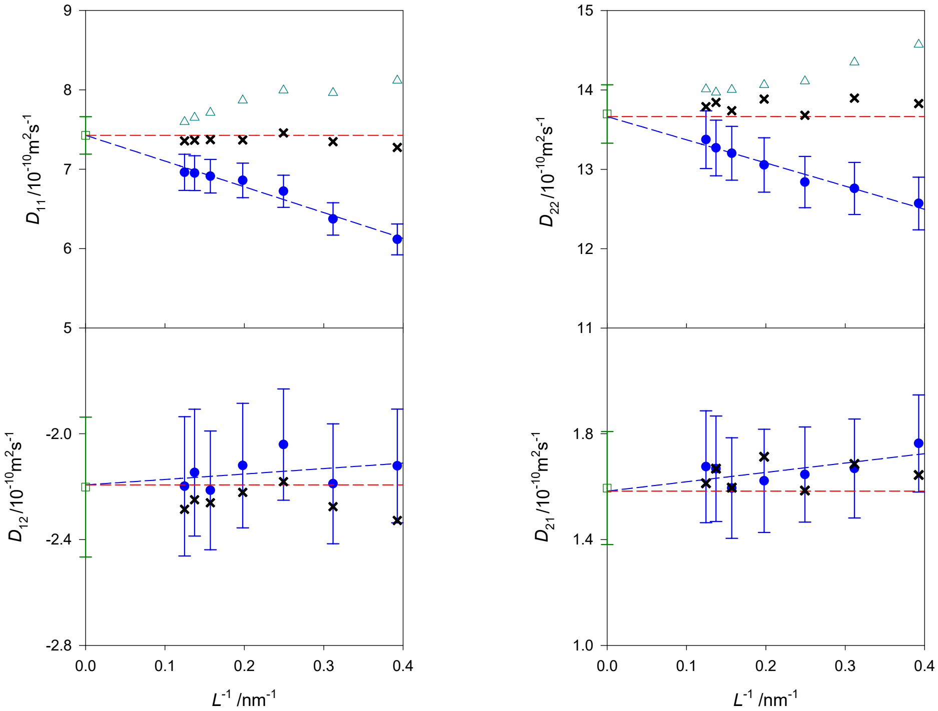

FIG. S16. Elements of the Fick diffusion coefficient matrix of the ternary subsystem water (1) + methanol (2) + 2-propanol (3) ($x_{\mathrm{H2O}} = 0.25$, $x_{\mathrm{CH4O}} = 0.5$ and $x_{\mathrm{C3H8O}} = 0.25$ mol·mol^{-1}) as a function of the inverse edge length of the simulation volume L^{-1} at 298.15 K and 0.1 MPa. The blue dashed line is a linear fit to the uncorrected simulation results (blue bullets). Coefficients subject to the fast correction procedure [11] (crosses) are compared with those corrected with the procedure by Jamali et al. [19] (cyan triangles). The green squares represent the Fick diffusion coefficients based on the individually extrapolated phenomenological coefficients.

REFERENCES

[1] X. Chen and Y. C. Hou, Determination of the density and viscosity for liquids and liquid mixtures, Gaoxiao Huaxue Gongcheng Xuebao **5**, 237 (1991).
[2] R. Taylor and R. Krishna, *Multicomponent Mass Transfer* (John Wiley & Sons, New York, 1993).
[3] J. L. F. Abascal and C. Vega, A General Purpose Model for the Condensed Phases of Water: TIP4P/2005, J. Chem. Phys. **123**, 234505 (2005).
[4] T. Schnabel, J. Vrabec, and H. Hasse, Henry's Law Constants of Methane, Nitrogen, Oxigen and Carbon dioxide in Ethanol from 273 to 498 K: Prediction from Molecular Simulation, Fluid Phase Equilib. **233**, 134 (2005).
[5] T. Schnabel, A. Srivastava, J. Vrabec, and H. Hasse, Hydrogen Bonding of Methanol in Supercritical CO2: Comparison between 1H-NMR Spestroscopic Data and Molecular Simulation Results, J. Phys. Chem. B **111**, 9871 (2007).
[6] Y. M. Muñoz-Muñoz, G. Guevara-Carrion, and J. Vrabec, Molecular Insight into the Liquid Propan–2–ol +Water Mixture, J. Phys. Chem. B **122**, 8718 (2018).
[7] G. Guevara-Carrion, J. Vrabec, and H. Hasse, Prediction of Self-diffusion Coefficient and Shear Viscosity of Water and its Binary Mixtures with Methanol and Ethanol by Molecular Simulation, J. Chem. Phys. **134**, 074508 (2011).
[8] G. Guevara-Carrion, C. Nieto-Draghi, J. Vrabec, and H. Hasse, Prediction of Transport Properties by Molecular Simulation: Methanol and Ethanol and their Mixture, J. Phys. Chem. B **112**, 16664 (2008).
[9] S. Parez, G. Guevara-Carrion, H. Hasse, and J. Vrabec, Mutual Diffusion in the Ternary Mixture of Water + Methanol + Ethanol and its Binary Subsystems, Phys. Chem. Chem. Phys. **15**, 3985 (2013).
[10] G. Guevara-Carrion, Y. A. Gaponenko, T. Janzen, J. Vrabec, and V. Shetsova, Diffusion in Multicomponent Liquids: From Microscopic to Macroscopic Scales, J. Phys. Chem. B **120**, 12193 (2016).
[11] G. Guevara-Carrion, R. Fingerhut, and J. Vrabec, Fick diffusion coefficient matrix of a quaternary liquid mixture by molecular dynamics, J. Phys. Chem. B **124**, 4527 (2020).
[12] R. Krishna and J. M. van Baten, The Darken Relation for Multicomponent Diffusion in Liquid Mixtures of Linear Alkanes: An Investigation Using Molecular Dynamics (MD) Simulations, Ind. Eng. Chem. Res. **44**, 6939 (2005).
[13] D. Alfe and M. J. Gillan, First-Principles Calculation of Transport Coefficients , Phys. Rev. Lett. **81**, 5161 (1988).
[14] G. Rutkai, A. Köster, G. Guevara-Carrion, T. Janzen, M. Schappals, C. W. Glass, M. Bernreuther, A. Wafai, S. Stephan, M. Kohns, S. Reiser, S. Deublein, M. Horsch, H. Hasse, and J. Vrabec, ms2: A Molecular Simulation Tool for Thermodynamic Properties, release 3.0, Comp. Phys. Commun. **221**, 343 (2017).
[15] R. Fingerhut, G. Guevara-Carrion, I. Nitzke, D. Saric, J. Marx, K. Langenbach, S. Prokopev, D. Celný, M. Bernreuther, S. Stephan, M. Kohns, H. Hasse, and J. Vrabec, ms2: A molecular simulation tool for thermodynamic properties, release 4.0, Comp. Phys. Commun. **262**, 107860 (2021).
[16] R. Lustig, Angle-average for the powers of the distance between two separated vectors, Mol. Phys. **65**, 175 (1988).
[17] M. Schoen and C. Hoheisel, The mutual diffusion coefficient D_{12} in binary liquid model mixtures. Molecular dynamics calculations based on Lennard-Jones (12-6) potentials, Mol. Phys. **52**, 33 (1984).
[18] M. P. Allen and D. J. Tildesley, *Computer Simulation of Liquids* (Clarendon Press, Oxford, 1987).
[19] S. H. Jamali, A. Bardow, T. J. H. Vlugt, and O. A. Moultos, Generalized form for finite-size corrections in mutual diffusion coefficients of multicomponent mixtures obtained from equilibrium molecular dynamics simulation, Chem. Theory Comput. **16**, 3799 (2020).
